# Effects of customer self-audit on the quality of maternity care in Tabriz: A cluster-randomized controlled trial

**DOI:** 10.1371/journal.pone.0203255

**Published:** 2018-10-11

**Authors:** Kamal Gholipour, Jafar Sadegh Tabrizi, Mohammad Asghari Jafarabadi, Shabnam Iezadi, Ahmad Mardi

**Affiliations:** 1 Iranian Center of Excellence in Health Management, School of Management and Medical Informatics, Tabriz University of Medical Sciences, Tabriz, Iran; 2 Health Services Management Research Center, Health Management and Safety Promotion Research Institute, Tabriz University of Medical Sciences, Tabriz, Iran; 3 Road Traffic Injury Research Center, Health Management and Safety Promotion Research Institute, Tabriz University of Medical Sciences, Tabriz, Iran; 4 Department of Statistics and Epidemiology, Faculty of Health, Tabriz University of Medical Sciences, Tabriz, Iran; 5 Social Determinants of Health Research Center, Health Management and Safety Promotion Research Institute, Tabriz University of Medical Sciences, Tabriz, Iran; 6 East Azerbaijan Provincial Health Center, Tabriz University of Medical Sciences, Tabriz, Iran; Queensland University of Technology, AUSTRALIA

## Abstract

**Objective:**

To study the effects of customer self-audit on the service quality (SQ) and customer quality (CQ) of maternity care.

**Design:**

A community-based cluster-randomized controlled trial.

**Setting:**

Twenty-one health centres and health posts in Tabriz, Iran.

**Participants:**

Of 21 health centres/health posts, 10 were randomly assigned to the intervention group and 11 randomly assigned to the control group. Participants were 185 pregnant women selected from health centre/post registration lists (intervention group: n = 92; control group: n = 93).

**Interventions:**

The intervention was a customer self-audit based on the CenteringPregnancy^®^ model of prenatal group care. The intervention group attended group support sessions focused on participants’ opinions, questions, and self-management concerns. They also received sessions on experiential learning, coping, problem-solving, and goal-setting by a family health expert, a midwife, and a doctor. Control group participants continued to receive individual care.

**Primary outcome measures:**

SQ and CQ were assessed using questionnaires. Patients rated the importance and performance of non-health quality dimensions. SQ was calculated as: SQ = 10 − (Importance × Performance).

**Results:**

Total mean SQ scores were 7.63 (0.91) and 8.91 (0.76) for the control and intervention groups, respectively, a statistically significant difference (p<0.001). Compared with the control group, the intervention group scored higher on the SQ aspects confidentiality, communication, autonomy, availability of support group, dignity, safety, prevention, and accessibility. Total mean CQ scores for the control and intervention groups were 82.63(7.21) and 87.47 (6.75), respectively, a statistically significant difference (p<0.001). After intervention, 82.6% of intervention group participants and 50.5% of control group participants reached the highest stage of self-management, showing an ability to take care of themselves under stress and financial constraints.

**Conclusions:**

The group prenatal care customer self-audit improved the SQ and CQ of maternity care by increased involvement of participants and giving them active roles in the care process.

## Introduction

Maternity care, a set of services to improve the health of both pregnant women and neonates, is essential in obtaining the desirable outcomes of prevention, detection, and treatment of complications [1, 2]. Such care has long been recognized as a means to identify high-risk mothers and provide a range of available medical, nutritional, and educational interventions to reduce the incidence of low birth weight and other adverse pregnancy conditions and outcomes [3, 4]. Prenatal care in Iran is traditionally practised through one-on-one encounters between a single pregnant woman and a single provider. Today’s maternity-related health education is an inseparable part of maternity care and empowers women to identify symptoms that could lead to potentially serious conditions and respond appropriately to them [1]. However, in some settings, maternity care has failed to attain pre-defined goals because of the low quality of services [5]. Low-quality care can increase standardized morbidity and mortality ratios [1, 6]. Therefore, quality improvement in maternity care is a high priority in many countries, particularly in low- and middle-income settings [6, 7]. An alternative model of prenatal care that has gained increasing attention for its efficiency and effectiveness is group prenatal care (GPC) [8]. GPC is an evidence-based approach to empower pregnant women and achieve patient-centred care by shifting from individual prenatal care visits to group care models [9–13]. Such models empower women by providing comprehensive care through group facilitation, education about self-management, group peer support, and focused discussions. Therefore, GPC has the potential to improve quality of care [9, 10].

Quality of maternity care is defined as a pregnancy and childbirth care in which the physical infrastructure, supplies and qualified human resources are available and delivery of care is performed within a normal physiological, social, and cultural processes. Quality of maternity care is a multidimensional concept [14] and here we refer to three dimensions of quality of maternity care namely: service quality (SQ), technical quality (TQ), and customer quality (CQ). TQ describes the clinical and technical aspects of care. The extent to which health care providers adhere to the standards is a reasonable indicator of the TQ of maternity care in health systems [15]. SQ refers to non-clinical aspects of care. SQ primarily describes how the received care is perceived and how it is influenced by the physical, social, and cultural context; it also includes non-health points of care, such as accessibility, respect, and confidentiality. CQ is based on service users or customer features and describes the extent to which customers obtain knowledge, skills, and confidence in their care process [16].

Focusing only on clinical or technical aspects of service fails to produce a comprehensive picture of quality of care. Therefore, understanding the customer’s perception of SQ and CQ can help providers to determine what their customers are seeking [17]. Assessment of customer perceptions of quality can help health care providers to fulfil their responsibilities to customers and increase customer satisfaction [18]. However, SQ and CQ have received little or no attention in quality improvement programs in many health care settings [19]. Doaee and colleagues have asserted that a consideration of women’s opinions is necessary to improve the quality of maternity services, as women are the service users and have useful information about the quality of services provided for them [20].

The SQ concept originated in Parasuraman’s (1988) work on service organizations. In 1991, SQ was redefined as a ‘multi-dimensional scale to capture customer perceptions and expectations of SQ which involves the calculation of the differences between expectations and perceptions on a number of specified criteria’ [21]. Different researchers in health care settings have proposed several aspects of SQ. For instance, Kumaraswamy (2012) highlighted five SQ aspects: reliability, responsiveness, assurance, empathy, and tangibility. In contrast, Tabrizi (2008) considered caring, access, and physical environment to be the core aspects of health SQ [21–23].

CQ is related to aspects of the customer, including knowledge, skills, and confidence. These qualities are essential for active participation in health care processes and decision making, performing appropriate activities, and changing the environment and health-related behaviours [16]. Most programs of quality improvement in developing countries that have used interventions such as in-service training, supervision, audit, and feedback, have focused on the interventions from the providers’ perspective [6]. The resulting neglect of the role of customers and their preferences in quality improvement initiatives may decrease the effect of such interventions on both health care quality and health outcomes [24]. The participation of women in making decisions about their health-related issues and patient-centred care, as well as the increased involvementof women, has positive effects on patient satisfaction and women’s self-confidence and emotional well-being. This engenders positive attitudes toward maternity care [25, 26]. A growing body of evidence suggests that the GPC model can improve satisfaction, outcomes, and effective delivery of care and enhance women’s capabilities by empowering them [27, 28]. Moreover, empowered women are able to actively participate in the care process and receive high quality care [11, 29]. Berwick et al. were the first researchers to emphasize the relationship between quality measurement and improvement, and the role of customer participation in quality measurement and improvement [30]. The customer self-audit is derived from the GPC and refers to a model of care in which the pregnant woman participates actively in the care process, is enabled to monitor the care process, and jointly participates with the care provider to guarantee adherence to standard care protocols and guidelines [15].

Tabrizi et al. have shown that the quality of non-clinical aspects of prenatal care in Tabriz is low. Pregnant women do not participate in decisions made about their care process, feel that they lack group support, and feel that there is little coordination with health care providers [31]. A low proportion of pregnant women in Tabriz believe that they play a critical role in the care process and only 14% are able to continue with prenatal care despite experiencing stress and financial constraints [16]. Although 69% of pregnant women in Tabriz receive six care visits during pregnancy, two-thirds do not receive standard, guideline-based care in these visits [32]. These studies show that customers who have received maternity care perceive that there is substantial room for quality improvement in most aspects of provided care, particularly support groups and safety [16, 31, 32]. To address such quality gaps, our team adapted the customer self-audit (which takes pregnant women’s views into consideration, addresses their need for support and communication, and enables them to actively participate in health care programs) to improve the quality of prenatal care in Tabriz. We assume that involvement of pregnant women in their care process and monitoring the maternity care by themselves will make them more confident and will encourage them to be more responsible for their care process. Thus, it is expected that customers’ request for high quality care push healthcare provider to meet their request in an acceptable quality [30]. The aim of this study was to examine the effect of the customer self-audit on the SQ and CQ of prenatal care in Tabriz.

## Materials and methods

A community-based cluster-randomized controlled trial was conducted to assess the effects of customer's self-audit in GPC on SQ and CQ of maternity care. The perspectives of pregnant women in Tabriz, Iran, were considered. The study was conducted in health centres and health posts in urban areas in Tabriz between September 2012 and May 2013. Primary health care in urban areas in Iran is provided in health centres and health posts. Health centres are the principal sources of care and provide care from physicians and health experts. Health posts provide care only from health experts and customers are referred to health centre physicians if necessary. Participants were recruited between September 14, 2012, and November 14, 2012. A follow-up was conducted between March 14, 2013, and May 14, 2013. The study design and procedure were approved by the Ethics Committee of Tabriz University of Medical Sciences before the study began. At the time of research proposal approval, clinical trial registration was generally not required for educational and non-medical clinical trials in Tabriz University of Medical Sciences, and the trial received approval by the local ethics committee. However, we registered the trial in 2013 when we prepared the research papers (registration number: IRCT2013020612378N1) to meet international guidelines. The authors confirm that all on-going and related trials for this drug/intervention were registered. Study participants were informed about the trial. After the briefing, participants gave written permission for their involvement.

### Randomization and blinding

**Fig 1:**
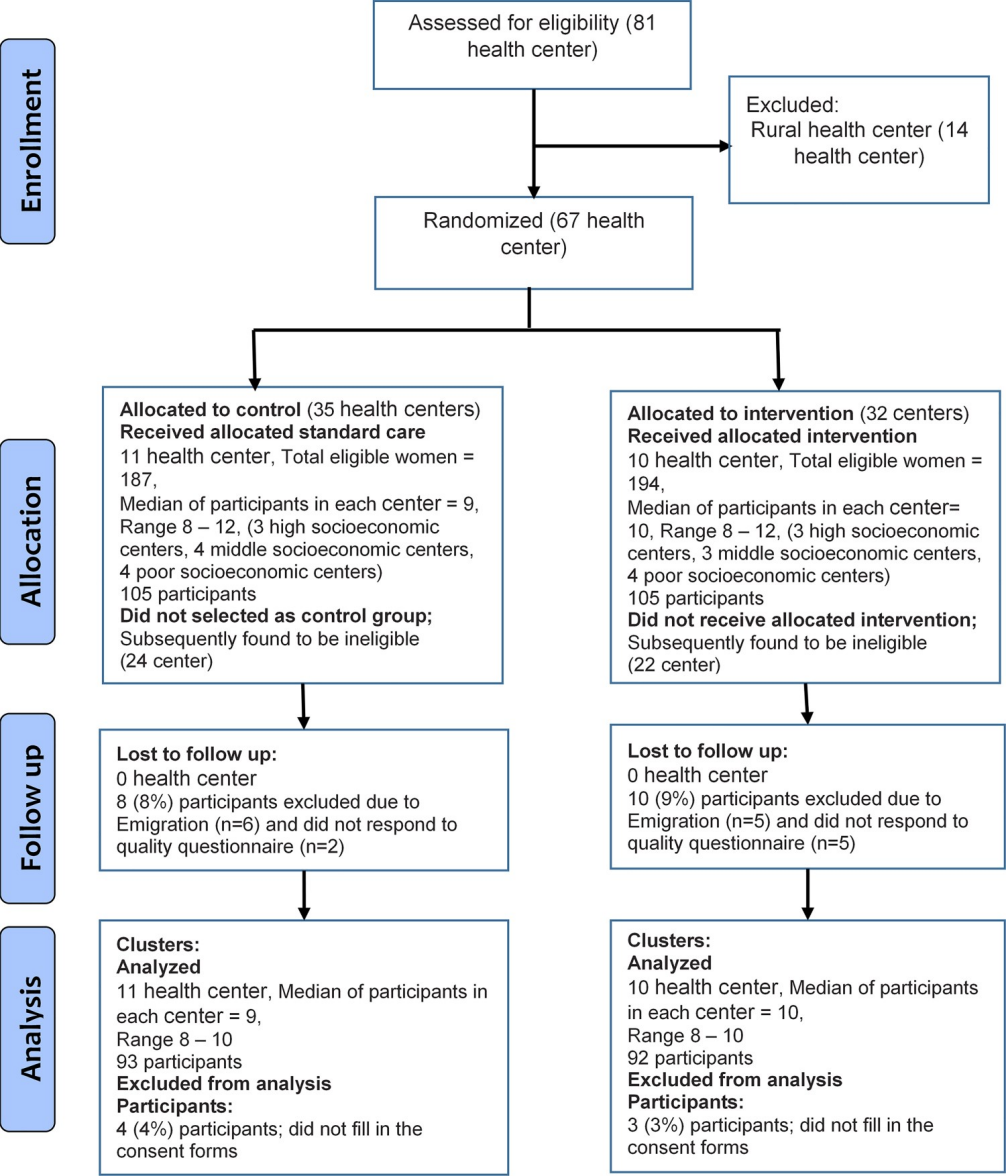
Eligibility and random allocation of study participants.

All 81 health centres/posts in the Tabriz urban area were stratified into three categories based on the socioeconomic status of the regions in which the centres/posts were located. Experts at the Family and Population Health Department of Tabriz University of Medical Sciences selected 21 health centres and health posts from registration lists of pregnant women. For this study, 6–8 centres in each category were randomly assigned to either an intervention or a control group; in total, 10 centres were allocated to the intervention group and 11 were allocated to the control group (Fig 1). Family health experts in the Vice-Chancellery for Health at Tabriz University of Medical Sciences generated a random allocation sequence, enrolled participants, and assigned them to intervention or control groups. Pregnant women who lived in Tabriz at the time of the study and were receiving health care services in health centres/posts of the public health system were included in the study. Women who had had less than three maternity care visits (in both the control and intervention group) and women who had declined to fill out the questionnaire by the final maternity visit before labour were not included in data analysis. Because of the nature of the intervention, blinding was not possible after assignment to interventions. However, data collection and data entry were carried out by two people who were neither members of the research team nor health care staff.

### Sample size

An 80% statistical power level and a 95% confidence level were used. G*Power version 3.1.2 (Franz Faul, Kiel University, Germany) [33], a statistical power analysis program, was used to calculate sample size. The maximum tolerable error rate was 5%. A standard deviation of 1 (established in our previous study [31]) was multiplied by a design effect of 1.5 (based on a study conducted in a provincial health centre). Assuming a 10% dropout rate, the sample size was calculated as 210; therefore, 96 individuals in each group were selected [34].

### Intervention

The customer self-audit was the intervention of interest and was based on an alternative model of prenatal group care termed the CenteringPregnancy® program, which includes group support and health education. The intervention group comprised 8–12 women who were all in the third month of pregnancy and who were randomly selected based on registration lists. Participants in the intervention group not only received national guideline-based care at individual visits, but also took part in self-care activities and attended group support sessions. Intervention group participants attended six sessions during pregnancy; each session lasted 1.5 h. The meetings focused on women’s opinions, questions, and concerns about self-management and their co-experiences of pregnancy challenges. In the sessions, women received educational tips about experiential learning, coping, problem-solving, and goal-setting from a family health expert, a midwife, and a doctor. At the beginning of each session, experts would address these issues and then participants would describe their experiences and discuss their problems, and the health experts would respond to them. At such sessions, participants not only received the education they needed, but also shared their experiences with each other. In addition, maternity books developed according to the maternity care protocols issued by the Iranian Ministry of Health [35] were provided to the intervention group. The books contained information about service standards during pregnancy, maternity educational material, and recommended care checklists for each meeting [36]. Health care providers taught participants how to use the books. The aim of the intervention was to inform women about their rights and standards of care during pregnancy, increased involvement of them to request standard services from their care provider, and remind them of what services they were entitled to. The assumption was that this process could ensure the quality of care.

Participants in the control group received only individual care in a treatment room from a midwife or doctor. Each visit lasted approximately 10 min. During each visit, participants received a routine check-up and examinations according to the national guideline. If participants had a particular problem, they discussed it with their midwife/doctor. A participant receiving only individual care could not share her experiences with other pregnant women and she received only the care recommended for her own situation. Control group participants did not receive information about service standards during pregnancy or pregnancy educational material. Control group participants were able to voluntarily attend one educational session, which was a lecture about breastfeeding, infant care, and delivery types (information about, and consequences of, normal delivery and caesarean section).

### Data collection

SQ data were collected using a structured questionnaire, which was based on the work of Van Der Eijk and modified to a maternity care setting [31, 37]. The content validity of the questionnaire was tested by 10 experts in maternity health care and quality of health care at Tabriz University of Medical Sciences. After being informed of the study objective, the experts reviewed and confirmed the validity of the questionnaire. Reliability was assessed using Cronbach’s alpha. Based on pilot data from 30 participants, Cronbach’s alpha was 0.85, and ranged from α = 0.70 for timeliness, to α = 0.93 for dignity (indicating moderate reliability). A structured questionnaire was used to gather data from both groups on CQ of maternity care. The face validity of the CQ questionnaire was reviewed and confirmed by the same 10 experts, and its reliability was confirmed using Cronbach’s alpha index (α = 0.803).

The study questionnaire had four main parts. The first part assessed demographic characteristics, the second part related to pregnancy condition, and the third part consisted of questions related to SQ. Twelve aspects of SQ were measured: *Choice of Care Provider*, choice of health centre, physician, and other providers (three questions); *Communication*, close relationship with physician/other provider and information sharing (five questions); *Autonomy*, participation in decision making about care procedures (four questions); *Support Groups*, support from other women, sharing information and experiences among women and professionals (four questions); *Continuity* (three questions); *Basic Amenities*, personnel and centre cleanliness and adequate amenities (four questions); *Dignity*, empathy, respect for beliefs and cultures, privacy, and providing accurate information to the patient’s family (five questions); *Timeliness*, (five questions); *Safety*, information about side effects of procedures and avoidance of harm to the patient (three questions); *Prevention*, information about high-risk pregnancy and self-care education (three questions); *Accessibility*, geographical and financial access (three questions), and *Confidentiality* (two questions). The fourth part of the questionnaire measured four stages of CQ related to maternity care: 1) Belief that the patient’s role is important, 2) Having the confidence and knowledge necessary to take action, 3) Actually taking action to maintain and improve one’s health, and 4) Continuing the self-management activities even under stressful conditions.

Participants were asked to evaluate the performance of each aspect of SQ according to either their understanding of the quality of care or their experience of receiving care that pertained to that aspect during the pregnancy. The importance of SQ was scored on a four-point Likert scale that ranged from 0 to 10: 0 = not important, 3 = may be important, 6 = important, and 10 = very important. Perceived quality of care received (performance) was assessed on a four-point Likert scale with the following categories: poor, fair, good, and excellent. Scores on this scale were dichotomized as 0 = good/excellent and 1 = poor/fair. A score was calculated for each SQ aspect by combining the importance and performance scores, based on the Quality of Care Through the Patient’s Eyes (QUOTE) scoring system instructions [37]. An overall SQ measure was also obtained. The aim of this method was to assess service performance by considering the importance of services from the customer’s perspective. Any SQ aspects that the customer did not consider important still received a performance score. Maternity care SQ was calculated according to the following formula: SQ = 10 –(Importance × Performance). Based on previous studies, SQ scores less than 9 indicated poor quality of care and a substantial opportunity for improvement [37]. SQ scores ranged from 0 (the worst) to 10 (the best). The SQ score for each aspect was obtained by calculating the average SQ score for that aspect’s questions. The total SQ score equalled the average SQ score for all 44 questions.

Data on the CQ of maternity care were collected using a structured questionnaire based on a study by Tabrizi et al. [16]. Raw CQ score was calculated by summing the responses to all 19 questions. Questions were rated using the following scale: strongly disagree = 1, disagree = 2, agree = 3, strongly agree = 4, and not applicable = 5. For responses of N/A or missing values (up to a maximum of three responses for each participant), the mean raw score was substituted [16]. Raw scores were normalized to 0–100 to calculate the active CQ scores. Active CQ scores were categorized to obtain customized cut-off points to identify the CQ score range for each stage of self-management [16, 38]. Based on this classification, women who achieved specified cut-off scores were considered to have passed that stage and were categorized into the next self-management stage (Table 1).

**Table 1:** Customer quality score cut-off points for self-management.

CQ scores	Self-management stage
19 and below	1. Belief in that the patients’ role is important
19.1 to 50	2. Having the confidence and knowledge necessary to take action
50.1 to 83	3. Actually taking action to maintain and improve one’s health
83.1 and above	4. Continuing the training course even under stress and financial constraints

### Outcome measures

Primary outcomes measures were the SQ and CQ of maternity care. SQ and CQ were assessed using questionnaires. Patients rated the importance and performance of non-health quality dimensions.

### Data analysis

Frequencies and percentages were used to summarize demographic information; means and standard deviations were used to summarize SQ and CQ scores. Chi-square and Fisher’s exact tests were used to analyse differences in categorical variables between groups. Independent samples t-tests were conducted to compare SQ scores between groups. To assess the effect of the intervention adjusted for planned pregnancy and education in CQ model and occupation, seeing midwife and effective maternity care in SQ model, a mixed model analysis was used, with the restricted maximum likelihood estimation method and first order autoregressive (RA (1)) covariance structure (taking into account the effect of sampling design). The P-values for entry and removal variables in the stepwise regression model were 0.05 and 0.10, respectively. P-values ≤0.05 were considered statistically significant. Data were analysed using the Statistical Package for the Social Sciences (SPSS) software for Windows, version 17 (SPSS Inc., Chicago, IL, USA).

## Results

Of 210 pregnant women who were contacted in health centres or health posts (105 participants in each group), 92 participants in the intervention group and 93 participants in the control group completed the study (86% response rate). Of the 25 non-responders, 11 (44%) people (5 in the intervention group and 6 in the control group) were excluded from the study because they had moved away from Tabriz, 7 (28%) were excluded owing to inability to answer the questionnaires, and 7 (28%) were excluded because they did not fill in the consent forms. The trial period ended after participants had delivered their babies.

Study participants in both groups were mostly aged between 20–30 years old (68%) and almost one-third (32%) had elementary and secondary education. Most participants (56.6% in the intervention and 53.8% in the control groups) were experiencing their first pregnancy; 73.9% of participants in the intervention group and 75.3% of participants in the control group had planned their pregnancy. Most of the participants in both groups (90.2% in the intervention and 87.1% in the control groups) were covered by health insurance (Table 2).

**Table 2:** Self-reported characteristics of study participants (intervention group, n = 92; control group, n = 93).

Characteristics		Intervention		Control		Chi-Square	P-value
		No.	%	No.	%		
**Age**	<20	12	13	9	9.7	0.84	
	20–30	63	68.5	63	67.7		0.656
	≥30	17	18.5	21	22.6		
**Education level**	Elementary and secondary	29	31.5	31	33.4	1.31	0.521
	High school	49	53.3	53	57		
	Tertiary	14	15.2	9	9.6		
**Number of pregnancies**	One	52	56.6	50	53.8	1.01	0.602
	Two	26	28.3	32	34.4		
	Three or more	14	15.2	11	11.8		
**Planned pregnancy**	Yes	68	73.9	70	75.3	0.04	0.832
	No	24	26.1	23	24.7		
**Occupation**	Employee	21	23	13	14	2.41	0.120
	Homemaker	71	77	80	86		
**Seeing midwife**	Yes	17	18.5	9	9.7	2.97	0.085
**Seeing obstetrician/gynaecologist**	Yes	58	63	65	69.9	0.97	0.324
**Having health insurance**	Yes	83	90.2	81	87.1	0.45	0.644
**Having continuity of care1**	Yes	91	99	89	96	1.82	0.368
**Effective maternity care2**	Poor and weak	2	2.2	7	6.6	2.86	0.091
	Good and excellent	90	97.8	86	93.4		

1 Seeing the same care provider for maternity care during pregnancy period.

2 Having overall effective maternity care during pregnancy period.

Of the different aspects of SQ, confidentiality and communication showed the highest score for both groups. The total SQ score was 8.91 (0.76) for the intervention group and 7.63 (0.91) for the control group (P<0.001). Compared with the control group, the intervention group scored higher on the SQ aspects of communication (P<0.001), autonomy (P = 0.023), availability of support group (P<0.001), dignity (P<0.001), safety (P<0.001), prevention (P<0.001), and accessibility (P = 0.038) (Table 3).

**Table 3:** Scores for aspects of SQ for the intervention and control groups (intervention group, n = 92; control group, n = 93).

*SQ*^*1*^ *aspect*	Group	Mean	SD	Mean Difference	95% Confidence interval		P-value
					Lower Limit	Upper Limit	
Choice of care provider	Inter^2^	8.78	1.40	-0.07	-0.47	0.32	0.711
	Cont^3^	8.85	1.32				
Communication	Inter	9.90	0.44	0.68	0.34	1.01	*>0*.*001*
	Cont	9.23	1.56				
Autonomy	Inter	9.33	1.77	0.56	0.08	1.04	0.023
	Cont	8.77	1.55				
Availability of support group	Inter	7.88	3.35	5.71	4.84	6.59	*>0*.*001*
	Cont	2.16	2.64				
Continuity of care	Inter	9.17	2.04	-0.09	-0.63	0.44	0.725
	Cont	9.27	1.60				
Basic amenities	Inter	8.67	2.11	-0.26	-0.79	0.26	0.325
	Cont	8.93	1.48				
Dignity	Inter	8.08	1.68	1.61	0.98	2.23	*>0*.*001*
	Cont	6.48	2.53				
Timeliness	Inter	8.19	1.87	0.29	-0.28	0.86	0.318
	Cont	7.90	2.07				
Safety	Inter	9.07	1.72	2.02	1.45	2.59	*>0*.*001*
	Cont	7.05	2.19				
Prevention	Inter	9.51	1.45	3.38	2.64	4.12	*>0*.*001*
	Cont	6.14	3.30				
Accessibility	Inter	9.07	1.62	0.51	0.03	1.00	0.038
	Cont	8.55	1.73				
Confidentiality	Inter	10.00	0.00	—	—	—	—
	Cont	10.00	0.00				
Total SQ score	Inter	8.91	0.76	1.28	1.04	1.52	*>0*.*001*
	Cont	7.63	0.91				

1 SQ: Service quality.

2 Inter: Intervention.

3 Cont: Control.

Generalized linear model analysis showed a statistically significant between-group difference in total SQ score: women in the intervention group had higher SQ scores (b = 1.24) (P<0.001) than those in the control group by adjusting for occupation, seeing midwife, and effective maternity care. Also, women who rated their received maternity care good and excellent had higher SQ scores (b = 1.07) (P = 0.007) than those who rated their received maternity care poor and weak (Table 4).

**Table 4:** Results of generalized linear model analysis SQ score (intervention group, n = 92; control group, n = 93).

Characteristics	B	Adjusted		P-value
		S.E.	Beta	
**Care group**				
Intervention	1.24	0.12	0.59	>0.001
Control*				
**Occupation**				0.223
Employed *				
home maker	0.21	0.17	0.08	
**Seeing midwife**				0.731
No*				
Yes	0.06	0.16	0.02	
**Effective maternity care**				0.007
Poor and weak *				
Good and excellent	1.07	0.38	0.22	

Dependent Variable: SQ.

* Reference Category.

SQ: Service quality.

B: Unstandardized coefficients.

S.E.: Standard error.

Beta: Standardized regression coefficients.

Goodness of Fit; Quasi Likelihood under Independence Model Criterion (QIC); (F change = 130.03, P>0.001).

### Self-Reported participant CQ scores

Analysis of the self-reported CQ scores showed that 50.5% of the participants in the control group achieved the highest score related to the self-management stages, but none of them believed in the importance of the patient role or rated themselves as having the confidence and knowledge necessary to take action. The results showed that 82.6% of participants in the intervention group continued their self-management activities, even under stressful conditions and financial constraints. The between-group difference in the distribution of participants in the self-management stages was statistically significant (P<0.001) (Table 5).

**Table 5:** Percentage of participants in each self-management stage.

Self-management Stages	intervention Group		Control Group		P value
	%	No	%	No	
1	-	-	-	-	<0.001
2	-	-	-	-	
3	17.4	16	49.5	46	
4	82.6	76	50.5	47	

### Relationship between CQ and participant characteristics

A univariate regression analysis of CQ scores showed that women who had planned their pregnancy had statistically higher CQ scores (P = 0.015). Study group (intervention or control) was significantly related to CQ score (P<0.001). Additionally, participants with higher educational status reported better CQ scores (Table 6).

**Table 6:** Relationship between CQ and participant characteristics (intervention group n = 92, control group n = 93).

Characteristics	CQ Mean (SD**)	Unadjusted			
		B	S.E.	Beta	P value
**Health Insurance**					
No*	83.79 (7.52)				
Yes	85.19 (7.36)	1.40	1.71	0.06	0.414
**Pregnancy History**					
One*	84.78 (6.74)				
Two	85.66 (8.37)	0.88	1.22	0.07	0.469
Three or more	84.63 (7.62)	-0.15	1.65	-0.01	0.929
**Occupation**					
Employed *	85.91 (6.1)				
home maker	84.84 (7.64)	-1.08	1.40	-0.06	0.444
**Continuous Care by Specialist**					
No*	84.37 (7.6)				
Yes	85.33 (7.29)	.96	1.18	0.06	0.418
**Planned Pregnancy**					
No*	82.79 (8.39)				
Yes	85.80 (6.86)	3.01	1.23	0.18	0.015
**Age**					
< 20	84.13 (8.88)	-0.96	2.01	-0.04	0.634
20–30	85.17 (6.85)	0.08	1.37	0.01	0.951
≥ 30*	85.09 (8.27)				
**Education**					
Elementary and Secondary*	83.01 (8.15)				
High school	85.76 (6.89)	2.75	1.18	0.19	0.021
Tertiary	87.11 (6.38)	4.10	1.78	0.18	0.023
**Group**					
Control*	82.63 (7.21)				
Intervention	87.47 (6.75)	4.85	1.03	0.33	<0.001

Dependent Variable: CQ (Customer Quality).

B: Unstandardized coefficients.

Beta: Standardized regression coefficients.

* Reference Category.

** Standard Deviation.

### Results of generalized linear model analysis of the relationship between CQ and participant characteristics

Generalized linear model analysis of total CQ scores showed a statistically significant between-group difference in total CQ score: women in the intervention group reported higher CQ scores (b = 4.81) (P<0.001) than those in the control group, by adjusting for education and planned pregnancy (Table 7).

**Table 7:** Results of generalized linear models for relationship between CQ and participants characteristics (intervention group, n = 92; control group, n = 93).

Characteristics	Adjusted			
	B	S.E.	Beta	P value
**Planned pregnancy**				
No*				
Yes	2.79	1.58	0.17	0.017
**Education**				
Elementary and secondary*				
High school	2.56	1.11	0.17	0.022
Tertiary	2.98	1.69	0.13	0.079
**Group**				
Control*				
Intervention	4.81	1.00	0.33	<0.001

Dependent Variable: CQ.

* Reference Category.

CQ: Customer quality.

B: Unstandardized coefficients.

S.E.: Standard error.

Beta: Standardized regression coefficients.

Goodness of Fit; Quasi Likelihood under Independence Model Criterion (QIC); (F change = 8316.53, P>0.001).

## Discussion

As an educational–informational intervention, the customer self-audit (based on GPC) had positive effects on the SQ and CQ of maternity care by increased involvement of participants through involving them in maternity care. Use of a customer self-audit in maternity care can help to improve perception of maternity care SQ, particularly the autonomy and communication aspects. Because of the nature of the intervention, we expected most improvement in the availability of the support group, safety, and prevention features, which are the SQ aspects most influenced by mothers’ knowledge of pregnancy care. However, the post-intervention improvement also encompassed other aspects of SQ, including autonomy, communication, dignity, confidentiality, and accessibility. The intervention group also showed higher scores on CQ of maternity care compared with the control group. Therefore, it can be concluded that customer self-audit can improve the CQ of maternity care from the perspective of pregnant women. Our findings support the recommendation of the World Health Organization that quality maternity care should be available to all pregnant women to assure a positive pregnancy experience [39]. GPC-based customer self-audit can provide a unified approach to maternity care in a group care setting that involves pregnant women in their care process, provides peer support, and prepares them by providing essential education [27]. Our results are similar to the positive findings of some previous studies on the effects of GPC on non-clinical aspects of maternity care, although in some cases the targeted outcomes were different to ours. For instance, a systematic review by Picklesimer and colleagues (2015) showed higher rates of breastfeeding and higher rates of participation in postpartum family planning among participants of GPC [40]. In another study, Cunningham and colleagues (2017) showed that GPC was effective for some requirements, such as providing services related to “high-touch” and “high-tech” issues, and could help to fulfil the triple aims of the health care system, one of which is better quality services [41]. The present findings indicated that the greatest between-group difference in SQ scores was for availability of a support group, communication, and autonomy. Regarding these three SQ aspects, the low score for the control group may have resulted from health centre structure, planning, and management. Although training by public health professionals, family health professionals, doctors, and midwives is very important, most centres did not have enough facilities for training. A joint publication by the World Health Organization and the United Nations Children’s Fund emphasized the need for community-based support groups, such as mother-to-mother support groups, and highlighted their effect on behaviour after childbirth, such as infant feeding [42]. Our results support the findings of a study in Taiwan (2004), which showed better SQ scores from group programs than from individual actions [43]. Several studies have also found that pregnant women who attend support group meetings have healthier pregnancies and report greater satisfaction with maternity care (especially in low-income settings) [10, 11, 29]. Accordingly, we can conclude that support groups facilitate improvement in other aspects of SQ. Although we found that the intervention group scored higher on autonomy, a study by Andersson (2013) showed that women in individual care experienced more autonomy than GPC participants [44].

The intervention group had significantly higher scores on the SQ aspects of safety and prevention during pregnancy. These findings support those of Mehdizadeh and colleagues (2003), who found that pregnant women who received health education and consultations, as well as training in neuromuscular exercises, were less tired and experienced less lumbar and pelvic pain during pregnancy compared with controls; they also were more physically active during the day than mothers who had not received such education and training [45]. Our findings showed that the intervention empowered women, as evidenced by the higher intervention group scores on the SQ aspect of dignity. This may be a result of customers’ increased awareness of their rights and the respect shown for the rights of service recipients in the care process. This aspect of SQ is closely associated with communication and interaction between clients and service providers. As mentioned above, pregnant women in the intervention group also scored higher on the SQ aspect of communication. A systematic review in 2013 reported high pregnancy satisfaction with GPC [46].

Confidentiality (i.e. ensuring the confidentiality of clients’ information) showed the highest SQ score in both intervention and control groups. This indicates that pregnant women consider this aspect of SQ important. The maintenance of confidentiality could also indicate whether health centres have reached optimal performance. In summary, the intervention had positive and significant effects on most aspects of SQ; however, there was no significant between-group difference for care provider, basic amenities, service continuity, and timeliness. This was partly predictable, because these areas are affected by organizational and infrastructural issues, and empowerment of providers and customers cannot influence these aspects.

The CQ scores demonstrated that all participants in both intervention and control groups passed self-management stages one and two. Moreover, all participants accepted that their role is important and that they have the necessary confidence and knowledge to take action, when it was needed, during the care process. Compared with the control group, significantly more women in the intervention group reached the highest stage of self-management. This indicates that, in addition to having the knowledge and ability to take action during the care process (because of the increased involvement), they could continue self-management activities even under stressful conditions and financial constraints. Our findings support those of several previous studies indicating that GPC has positive results. For instance, a study by Patil et al. (2017) showed that GPC was strongly associated with higher pregnancy-related empowerment in Malawi [47]. Kennedy et al. showed that women gain more knowledge and self-confidence during GPC, and therefore become more accepting of the physical changes they are experiencing and more engaged with the health care system [48]. Another qualitative study showed that a combination of additional time for a provider-delivered curriculum, open group question-and-answer time, and the opportunity to learn from other women resulted in substantially greater stress reduction, confidence enhancement, knowledge improvement, motivation, and informed decision making for group members [49]. Andersson et al. found that GPC helps to establish social contacts among women, which encourages them to care about one another’s health [44]. Herrman et al. found that the support and encouragement in GPC can help women in difficult conditions, such as those under stress or in vulnerable families [50].

The present findings revealed that having a planned pregnancy and higher education are predictors of higher CQ. This indicates the need for greater focus on women with lower educational status and unwanted and unplanned pregnancies. Joshi et al. and Neupane et al. found that less educated women from socioeconomically disadvantaged households require more attention and greater help [51, 52]. In addition, Koch et al. showed that greater educational levels have a positive effect on maternal mortality rates, on the access to, and utilization of, maternal health facilities, and on changes in women’s reproductive behaviour [53]. Regarding the importance of the group care model, it should be noted that mothers’ experiences of group activities to date has mainly included either antenatal training activities or new mothers’ groups. Recently however, there has been an increasing emphasis on the importance of GPC, which improves social support and information sharing [54]. GPC is welcomed by women and can lead to the best use of midwives’ time, improvement in social networks, and increased pregnancy-related knowledge [48, 55].

Although several studies have evaluated whether GPC can improve the quality of care, few studies have used self-audit as a model. Participants in an Australian CenteringPregnancy® study reported supportive relationships between women and their peers or midwives as one of the strengths of GPC. Participants in this study recorded increased satisfaction from the care they received [54]. In two different studies, Massey and colleagues (2006) and Jafari (2010) showed that women who took part in GPC supported each other during the prenatal care process and took responsibility for their own health during pregnancy. Furthermore, they helped to maintain and improve one another’s health [56, 57]. Gennaro and colleagues (2016) showed that providing support for pregnant women and incorporating knowledge and skills through maternity care may promote both physical and mental health in minority women [58].

The present findings have some generalizability, because the health centres were selected randomly and the features of these centres are similar to others in both Tabriz and other public health centres in Iran. As GPC is feasible to implement and produced positive results from the customer self-audit, we suggest that this intervention should be provided to all pregnant women in Iran. A focus on improving CQ as an indicator of customer capabilities may be an effective strategy to improve other dimensions of care quality, such as SQ and TQ. Such improvements may positively affect the health of mothers and their babies, and have other health consequences. The current study showed that pregnant mothers not only require traditional individual care from health care services, but also need to share their experiences with other pregnant women, feel that they are not alone, be involved in their care process, and audit their received services. However, there is a pressing need to widen this research to rural areas. More researchers are needed to evaluate the cost-effectiveness of customer self-audit in GPC and its effects on clinical outcomes.

### Limitations

This study had some limitations. The first is that only urban health centres were included. Therefore, the generalizability of results to rural areas is limited, as the socioeconomic characteristics of individuals in urban areas are different from those in rural areas. The second limitation is lack of blinding. Because of the nature of the intervention, it was not possible to blind participants. However, blinding was used in the data gathering and data evaluation, as these processes were conducted by an individual not involved in the implementation of the intervention. The last limitation relates to lack of health centre facilities to fully implement the intervention; to carry out GPC adequately, health centres must be able to host meetings.

Some of the strengths of our study were the high validity of the results owing to the study design, the use of valid and reliable questionnaire to assess maternity care SQ and CQ, and the low-cost intervention design.

## Conclusions

An intervention in response to the low quality of maternity care in Tabriz, the customer self-audit, was effective in improving the quality of non-health aspects of maternity care. The intervention increased involvement of pregnant women by providing active roles for them in the care process and increasing their awareness of their rights. Increasing mothers’ awareness of their rights and involving them in monitoring the care process had a positive effect on their perceptions and expectations of the quality of services. In addition, women who were involved in the customer self-audit were more likely than women in the control group to be able to take care of themselves under stressful conditions and financial constraints. The finding that educational status and planned pregnancy had a positive effect on CQ indicate the importance of focusing on improving care for less educated women and increasing the access to, and utilization of, family planning and pre-pregnancy services in the health system. The results of this study will be used either in national level by policymakers or in local level by healthcare providers to revise the way in which maternity care is provided for pregnant women in Iran. In developing countries like Iran, where the maternity care is delivered in a traditional manner (individual maternity care visit) a shift to a more participatory method that involves pregnant women in their care process will result in more satisfaction regarding nonclinical aspects of care. This study introduces such a low-cost alternative for traditional maternity care.

## Supporting information

S1 File Data. (RAR)

S2 File English language ethical letter. (PDF)

S3 File Original ethical letter. (JPG)

S4 File Research proposal approval letter. (PDF)

S5 File Original study protocol. (PDF)

S6 File English language protocol. (PDF)

S7 File Measures. (PDF)

S8 File Original study questionnaire. (PDF)

S9 File CONSORT checklist. (PDF)
