# Supplementary material for: Effects of customer self-audit on the quality of maternity care in Tabriz: A cluster-randomized controlled trial
Source: PLoS One. 2018 Oct 11;13(10):e0203255. doi: 10.1371/journal.pone.0203255 (PMC6181295; doi:10.1371/journal.pone.0203255)
Supplement: S2 File — (PDF) [file pone.0203255.s002.pdf]

Date: March 16, 2012.
Number: 5/4/12323

Deputy of Research and Technology

Research and Technology Deputy of University

Research proposal titled "improving quality of prenatal care from pregnant women's viewpoints in Tabriz" have been approved in 131th ethic session in March 11, 2012.

Dr.Alireza Ostadrahimi
Research Executive

Attar Nishabouri Rd
Golgasht
Tabriz, Iran
Deputy of Research and Technology
2th Floor, #2 Central Building
Phone: +98(41) 33357310
Fax: +98(41) 33344280
www. http://tbzmed.ac.ir/research
