# Supplementary figures and images for: Effects of customer self-audit on the quality of maternity care in Tabriz: A cluster-randomized controlled trial
Source: PLoS One. 2018 Oct 11;13(10):e0203255. doi: 10.1371/journal.pone.0203255 (PMC6181295; doi:10.1371/journal.pone.0203255)

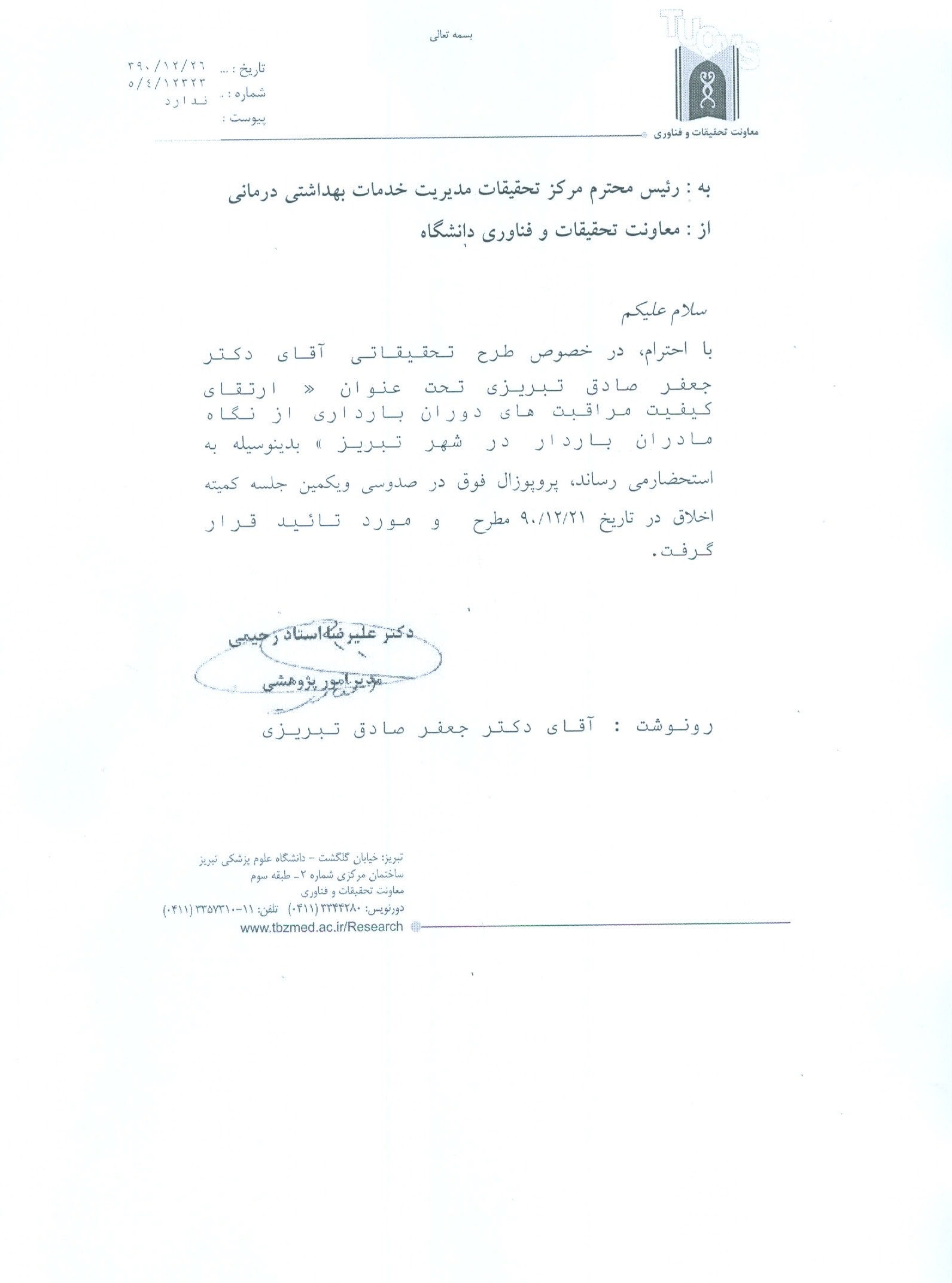

Supplement: S3 File — (JPG) [file pone.0203255.s003.jpg]
