# Supplementary material for: Effects of customer self-audit on the quality of maternity care in Tabriz: A cluster-randomized controlled trial
Source: PLoS One. 2018 Oct 11;13(10):e0203255. doi: 10.1371/journal.pone.0203255 (PMC6181295; doi:10.1371/journal.pone.0203255)
Supplement: S4 File — (PDF) [file pone.0203255.s004.pdf]

تاریخ: 12-Sep-2012
شماره: 5/77/2741
پیوست: No Attachment

دانشگاه علوم پزشکی و خدمات بهداشتی درمانی تبریز
مرکز تحقیقات مدیریت خدمات بهداشتی درمانی تبریز
مرکز کشوری مدیریت سلامت
WHO Collaborating Center for Training and Research on Health Management

To: Deputy of Research and Technology School of Management and Medical Informatics

Subject: Approval of the Dr Tabrizi projects

Greetings and Regards

In response to a number 3167/91/6 dated 12-Sep-2012 notifies that the Dr Jafar Tabrizi research project entitled "**Improving quality of maternity care from the perspective of pregnant women in Tabriz**" in 113th session of the Research Council and the Centre 124th session of the university ethics committee approved and the contract was signed.
The total budget of this project is equal to 97 million Rials (equal to 2770 US$) after deducting 10% tax, to cover conducting project costs. It is expected at least 3 JCR indexed articles to be published based on the result of this project. The code of ethics is TBZMED.REC. 5/4/12323 plan.

دکتر جعفر صادق تبریزی
رئیس مرکز تحقیقات مدیریت خدمات بهداشتی درمانی

آدرس ۱: تبریز، چهارراه عباسی، بلوار شهید رجایی، خیابان ۲۴ متری شهید شفیع زاده
آدرس ۲: تبریز، بزرگراه پاسداران، خروجی توانیر میدان ولی امر، خیابان ۲۴ متری شهید شفیع زاده
تلفن: ۰۴۱۳ ۶۵۷۱۸۱۳ - فکس: ۰۴۱۳ ۶۵۷۸۹۷۴ - کد پستی: ۵۱۵۵۶۶۸۴۷۴
پست الکترونیک: hmrc@tbzmed.ac.ir وب سایت : http://hmrc.tbzmed.ac.ir
پست الکترونیک: npmc@tbzmed.ac.ir وب سایت : http://npmcweb.tbzmed.ac.ir
