# Supplementary material for: Effects of customer self-audit on the quality of maternity care in Tabriz: A cluster-randomized controlled trial
Source: PLoS One. 2018 Oct 11;13(10):e0203255. doi: 10.1371/journal.pone.0203255 (PMC6181295; doi:10.1371/journal.pone.0203255)
Supplement: S5 File — (PDF) [file pone.0203255.s005.pdf]

بسمه تعالی کد:

معاونت پژوهشی

# پرسشنامه طرح تحقیقاتي

---

نام و نام خانوادگی: دکتر جعفر صادق تبریزی

عنوان طرح: ارتقای کیفیت مراقبت های دوران بارداری از نگاه گیرندگان خدمت در شهر تبریز

چنانچه نتیجه نهائی این طرح ،به تدوین و انتشار مقاله ای منجرشود ذکر منبع تامین مالی (معاونت پژوهشی دانشگاه علوم پزشکی تبریز ) ضروری می باشد .

---

نشانی: تبریز – دانشگاه علوم پزشکی تبریز – سازمان مرکزی شماره ۲ – معاونت پژوهشی-تلفن ۱۴-۳۳۵۷۳۱۳

## ۱- نام طرح:

الف) عنوان به فارسی:

**ارتقای کیفیت مراقبت های دوران بارداری از نگاه مادران باردار در شهر تبریز**

ب) عنوان به انگلیسی:

**Improving quality of maternity care from the perspective of pregnant women in tabriz.**

۲- طرح تحقیقاتی حاضر در راستای کدام یک از اولویت های پژوهشی دانشگاه (پیوست ) می باشد.(عنوان اولویت و زیر مجموعه مربوطه)

## ۳- اطلاعات مربوط به مجری و محل اجرای طرح :

| | |
|---|---|
| نام و نام خانوادگی | جعفر صادق تبریزی |
| نشانی و تلفن محل کار | دانشکده بهداشت و تغذیه ؛ گروه مدیریت وبهداشت عمومی |
| شغل و سمت فعلی | عضو هیئت علمی ، استادیار |
| رشته تحصیلی و تخصصی | PhD مدیریت خدمات بهداشتی و درمانی |
| محل یا محل های اجرای طرح | مراکز و پایگاه های بهداشتی شهر تبریز |
| شماره حساب / بانک ملی شعبه دانشگاه | ۰۳۰٤۲۳٦۰۸۹۰۰۸ |
| تلفن همراه | ۰۹۱٤٤۰۸٥٤۲۰ |
| **E-mail** | tabrizijs@tbzmed.ac.ir |

## ٤- مشخصات افراد

| نام و نام خانوادگی | شغل | درجه علمی و رشته تحصیلی | نوع همکاری* | کل ساعات کار | حق الزحمه در ساعت (ریال) | جمع (ریال) | امضا |
|---|---|---|---|---|---|---|---|
| مجری و همکاران اصلی | | | | | | | |
| جعفر صادق تبریزی | عضو هیئت علمی _ استادیار | PhD مدیریت خدمات بهداشتی و درمانی | نوشتن پروپوزال، طراحی پرسشنامه، طراحی مداخله، تجزیه و تحلیل دادهای قبل و بعد از مداخله، نوشتن گزارش و مقاله | ۵۰۰ | | | |
| مصطفی فرحبخش | مدیر IT مرکز بهداشت استان | دکترای پزشکی عمومی، همطراز هیئت علمی | نوشتن پروپوزال، طراحی پرسشنامه، طراحی مداخله، نوشتن گزارش و مقاله | ۳۵۰ | | | |
| دکتر مردی | | دکترای پزشکی عمومی | طراحی مداخله، جمع آوری داده های مداخله | ۱۰۰ | | | |
| مجتبی محمدزاده | | دکترای پزشکی عمومی | طراحی مداخله، جمع آوری داده های قبل و بعد از مداخله، نوشتن گزارش و مقاله | ۲۵۰ | | | |
| خانم افشار نیا | کارشناس مسئول بهداشت خانواده مرکز بهداشت شهرستان تبریز | کارشناس بهداشت | طراحی مداخله، آموزش کارکنان بهداشت خانواده، آموزش در گروه های حمایتی، جمع آوری داده های مداخله | ۲۰۰ | | | |
| خانم فرشباف | کارشناس بهداشت خانواده مرکز بهداشت استان | کارشناس بهداشت | طراحی مداخله، آموزش کارکنان بهداشت خانواده، آموزش در گروه های حمایتی، جمع آوری داده های مداخله | ۲۰۰ | | | |
| محمد اصغری | عضو هیئت علمی _ استادیار | PhD آمار | مشاوره آماری در پروپوزال، کمک به تحلیل داده های مداخله | ۱۰۰ | | | |
| کمال قلی پور | دانشجو | کارشناسی ارشد مدیریت خدمات بهداشتی درمانی | نوشتن پروپوزال، جمع آوری و وارد کردن داده های قبل و بعد از مداخله، آنالیز داده ها، نوشتن گزارش و مقاله | ٤۰۰ | | | |
| شبنم ایزدی | دانشجو | کارشناسی ارشد مدیریت خدمات بهداشتی درمانی | نوشتن پروپوزال، جمع آوری و وارد کردن داده های قبل و بعد از مداخله، آنالیز داده ها، نوشتن گزارش و مقاله | ۲۰۰ | | | |
| رحیمه علیپور | دانشجو | کارشناسی مدیریت خدمات بهداشتی درمانی | جمع آوری و وارد کردن داده های قبل از مداخله، آنالیز داده ها، نوشتن گزارش و مقاله | ۲۰۰ | | | |
| کارکنان مراکز بهداشتی | کارکنان مراکز بهداشتی شهرستان تبریز | کارشناس بهداشت عمومی | آموزش، ارائه مراقبت های دوران بارداری، جمع آوری داده | ۱۰۰۰ | | | |
| | | | | جمع هزینه (ریال) | | | |

* نوع همکاری، بطور دقیق و به تفکیک برای هر یک از افراد در ارتباط با اجرای طرح، ذکر گردد.

## ۵- مقدمه و بیان مسئله :( طبق بند ۱و۲ راهنمای تکمیل پرسشنامه )

آنچه که مسلم است در دنیای امروزی، به ویژه در کشور های در حال توسعه، به لحاظ محدودیت های منابع (مالی، انسانی، تجهیزاتی و ...) و همچنین سرعت ظهور تکنولوژی های جدید، افزایش فزاینده هزینه های تکنولوژی های نو و از سوی دیگر افزایش آگاهی و دانش گیرندگان خدمات سلامت نسبت به آنچه که باید دریافت نمایند و افزایش انتظارات به حق آنها برای دریافت خدمات با کیفیت بالا، ارتقای مستمر کیفیت خدمات دیگر یک انتخاب برای مدیران عرصه سلامت و سیستم ها نمی باشد بلکه یک امر اساسی است برای حیات سیستم ها.

در راستای تایید مطالب فوق منابع علمی ثابت کرده اند که ارتقای کیفیت خدمات نه تنها موجب ارائه خدمات بهینه به مشتریها (گیرندگان خدمات) می شود بلکه به طور چشم گیری باعث افزایش رضایتمندی، تمایل به مراجعات بعدی، کاهش در زمان صرف شده برای دریافت خدمت مورد نظر شده و از سوی دیگر موجبات کاهش هزینه ها برای سیستم را فراهم میسازد. شواهد فوق حاکی از این واقعیت است که ارتقای کیفیت خدمات ارائه شده در واحدهای بهداشتی درمانی باید یکی از اهداف بنیادین و الویت های اساسی سیستم های سلامت و مدیران سیستم ها باشد (1 - 3).

با توجه به شرایط ذکر شده مدیران باید ارتقای کیفیت را در سرلوحه کارهای خود قرار داده و آن را به یک فرهنگ عمومی جهت مشارکت همه کارکنان در تمام سطوح سازمان مبدل سازند، به طوریکه تمامی کارکنان در هر واحدی که مشغول فعالیت هستند ضمن شناسائی فرایند های حوزه فعالیت خویش، مشتری ها و تدارک کنندگان فرایندها، برنامه منظمی برای ارتقای فرایند های حیطه خود بر اساس اولویت ها را داشته باشند. بدیهی است مشارکت مشتری ها، به عنوان عضو موثر تیم های سلامت، در بهبود کیفیت خدمات از اهمیت شایان توجهی برخوردار است و نیازمند توجه ویژه تمامی مدیران و کارکنان سیستم می باشد.

با توجه به اهمیت ارتقای کیفیت خدمات در نظام سلامت، روش اندازه گیری کیفیت خدمات و اندازه گیری جامع آن می تواند از اهمیت افزونتری برخوردار باشد. امروزه از روش ها و مدل های مختلفی برای اندازه گیری کیفیت خدمت استفاده میشود، برای مثال در سال ۱۹۸۰ دونابدین (4) ، یکی از پیشگامان ارتقای کیفیت در خدمات بهداشتی درمانی مدلی مرکب از ۱) کیفیت فنی ، ۲) کیفیت ساختار و ۳) کیفیت فرایند را برای سنجش کیفیت خدمات ارائه داد که برای یک مدت طولانی مورد استفاده محققین در سراسر دنیا قرار گرفت. مدل فوق در سال 1999 توسط Kenagy و Berwick (5) اصلاح و تکمیل گردیده و به عنوان مدل موثری برای اندازه گیری کیفیت خدمات بهداشتی درمانی معرفی

گردید که شامل ابعاد ۱) کیفیت فنی ( Technical Quality) و ۲) کیفیت خدمت (Service Quality) می شد. مدل فوق نیز از زمان ارائه تا به امروز توسط محققین ارتقای کیفیت در نظام سلامت به طور مکرر استفاده شده و نتایج آن در مقالات مختلف علمی منعکس گردیده است.

براساس تعاریف ذکر شده توسط Kenagy و Berwick (۵) کیفیت فنی آنچیزی است که مشتری دریافت می کند در مقایسه با آن چیزی که بر اساس شواهد علمی به عنوان استاندارد خدمت معرفی شده است. این بعد از کیفیت غالبا" انعکاسی است از دانش ، مهارت و توانمندی های ارائه کننده خدمت. از سوی دیگر کیفیت خدمت بر خلاف کیفیت فنی به شرایط و چگونگی دریافت خدمت توسط مشتری (گیرنده خدمت) مربوط بوده و اغلب نشان دهنده روش ارائه خدمت و محیطی است که خدمت یاد شده در آن ارائه میشود.

با توجه به اینکه هر دو مدل ذکر شده در بالا کیفیت خدمت ارائه شده را از بعد اختصاصی (کیفیت فنی) و بعد غیر مرتبط با سلامت (کیفیت خدمت) می سنجند، به نظر میرسد بعد بسیار مهم و حیاتی در ارائه خدمات بهداشتی درمانی به غفلت سپرده شده است. این بعد مربوط به گیرنده خدمت، دانش او در قبال خدمتی که دریافت میکند، ویژه گیها و توانمندیهای مشتری، که بدون تردید تاثیر چشم گیری بر نحوه ارائه و دریافت خدمت دارد را شامل می شود. قابلیت و توانمندیهائی که باعث مشارکت هر چه بیشتر و بهتر در فرایند ارائه خدمت، مداخله در موارد ضروری، دخالت در تصمیم گیری های کلیدی، تعامل سازنده با ارائه کننده خدمت و ...را سبب میشود. با توجه به ضرورت توجه به نکات بالا در این طرح تحقیقاتی از مدل Comperhensive Quality Measurment in Health care (CQMH) که توسط تبریزی و همکاران (٦) در سال ۲۰۰۷ ارائه شده است برای سنجش جامع کیفیت استفاده خواهد شده که در آن بعد کیفیت مشتری ( Customer Quality) به ابعاد قبلی اضافه شده است. کیفیت مشتری مجموعه ویژه گیها و توانمندیهائی است که گیرنده خدمت برای مشارکت موثر در فرایند خدمات بهداشتی درمانی، تصمیم گیری های کلیدی و مداخلات صحیح و به موقع به آنها نیازمند است.

در این طرح تحقیقاتی کارائی مدل CQMH در اندازه گیری کیفیت مراقبت ها و خدمات پیشگیری، و به طور اختصاصی در خدمات ارائه شده به زنان باردار در طول ۹ ماه بارداری مورد بررسی قرار می گیرد. چرا که سلامت زنان باردار یکی از مهم ترین و اساسی ترین نیاز های بهداشتی هر جامعه ای می باشد. با وجود چنین اهمیتی هر ساله حدود ۹۹٪ از ۵۲۹۰۰۰ مرگ مادران ناشی از بارداری و ۹۸٪ از ۵،۷ میلیون مرگ ناشی از زایمان در کشور های در حال توسعه اتفاق می افتد (۷). تخمین زده می شود زنان در کشور های در حال توسعه ۱٤۰ بار بیشتر در معرض خطرات ناشی از بارداری قرار دارند. دراهداف توسعه هزاره (هدف ۳ و ٤ و۵) توجه به سلامت ورفاه زنان از شاخص های اصلی توسعه ی جوامع مطرح شده و هدف ۵ ارتقاء سلامت زنان باردار را مطرح وآن را جزء ۸ هدف اصلی توسعه می داند ( 8). سلامت مادران باردار ابعاد مختلفی داشته و مولفه های گوناگونی در دستیابی به این هدف دخیل هستند که از آن جمله می توان به موارد زیر اشاره کرد: دسترسی به بهورزان آموزش

دیده، آگاهی اجتماعی در باره ی نیاز های سلامت بارداری، ارتقای سطح سلامت زنان، ارتقای عملکرد ارائـه کننـدگان خـدمات بهداشـتی، ایجـاد سیستم پایش میزان مرگ و میر مادران، هدایت و سامان دهی خدمات ماماهای سنتی، افزایش بهره وری تسهیلات موجود و توسـعه سیاسـت هـای استراتژیک مناسب برای ارتقای کیفیت مراقبت های بارداری، زایمان ،تنظیم خانواده و تسهیلات زایمانی.

مراقبت های بارداری به مجموعه ای از خدمات نظام مند اتلاق می شود که طی دوران بارداری مادر را از لحاظ وضعیت سلامت پایش و زمینـه را برای تولد نوزادی سالم آماده میکند. تحقیقات نشان می دهند که مراقبت های دوران بارداری میزان مرگ و میر مادران و نوزادان را کـاهش داده و سطح سلامت مادران و نوزادن را افزایش می دهد. مراقبتهای دوران بارداری یکی از ارزشمند ترین و اقتصادی ترین برنامه های بهداشتی هستند که نتایج کاملا مشهودی بر وضعیت سلامت داشته و از تحمیل هزینه های اضافی بر دوش سیستم سلامت مـی کاهـد(۹). بیشـتر مطالعـات ، کفایـت مراقبت های قبل از تولد را بر اساس زمان و تعداد مراقبت ها تعیین می کنند و توجهی به محتوای خدمات ندارند. در صورتی کـه محتـوای یـک ویزیت قبل از تولد به همان درجه تعداد و دفعات مراقبت اهمیت دارد . البته کم بودن تحقیقـات کیفـی درایـن زمینـه، احتمـا ً ناشـی از فقـدان معیارهای واحد و استاندارد که قابلیت اشتراک دربین کشورها را داشته باشد ناشی می گردد (۱۱و۱۰). از آنجائیکه نتایج مطالعـات انجـام شـده در کشورهای پیشرفته مؤید این نکته است که ارائه مراقبت های با کیفیت بالا در دوران بارداری، به عنوان یک مداخله مؤثر موجب کاهش مرگ و میر شیرخواران (Infant Mortality Rate-IMR)، کاهش مرگ و میـر مـادران ناشـی از زایمـان و بـارداری-Maternal Mortality Rate) (MMR و بخصوص کاهش مرگ و میر حول زایمان (Perinatal Mortality Rate-PMR)، ضرورت توجه به مراقبت های دوران بارداری، پایش، ارزیابی و بهبود کیفیت این مراقبت ها وپاسخ به نیاز ها و انتظارات مادران باردار اهمیت افزونتری پیدا میکند(۱۲،۱۳،۱٤) .

تحقیقات نشان می دهند که ارتباط تنگاتنگی بین کیفیت مراقبت های دوران بارداری و آگاهی و نگرش مادران دریافت کننده این مراقبت ها وجود دارد. بطوریکه هر اندازه این مولفه ها در فرد بالا باشد احتمال دستیابی به خدمات و مراقبت های مناسب و مطلوب نیز افزایش می یابـد. همچنـین تحقیقات انجام شده نشان می دهد که میزان رضایت مادران از کیفیت اطلاعات ارائه شده در باره ی مراقبت های دوران بـارداری در سـطح پـایینی قرار دارد که این امر یکی ازمشکلات پیش رو دردستیابی به اهداف مراقبت ها و ارتقای کیفیت مراقت ها می باشد (۱٦و۱۷و ۱۵).

**٦- تعریف واژه های اختصاصی**

کیفیت خدمت (Service Quality) : کیفیت خدمت به شرایط و چگونگی دریافت خدمت توسط مشتری (گیرنده خدمت) مربوط بوده و اغلب نشان دهنده روش ارائه خدمت و محیطی است که خدمت یاد شده در آن ارائه میشود (٦).

کیفیت فنی ( Technical Quality) : آنچه که مشتری دریافت می کند در مقایسه با آن چیزی که بر اساس شواهد علمی به عنوان استاندارد خدمت معرفی شده است. این بعد از کیفیت غالبا" انعکاسی است از دانش ، مهارت و توانمندی های ارائه کننده خدمت (۵).

کیفیت مشتری ( Customer Quality) : مجموعه ویژه گیها و توانمندیهائی است که گیرنده خدمت بـرای مشـارکت مـوثر در فراینـد خـدمات بهداشتی درمانی، تصمیم گیری های کلیدی و مداخلات صحیح و به موقع به آنها نیازمند است (٦).

## ۷- فرضیات طرح :

۱. از دیدگاه مشتریها (گیرندگان مراقبت های دوران بارداری)، وضعیت کیفیت فنی بعد از انجام مداخله بین گروه‌های تجربی و شاهد اختلاف دارد.

۲. از دیدگاه مشتریها (گیرندگان مراقبت های دوران بارداری)، وضعیت کیفیت خدمت بعد از انجام مداخله بین گروه‌های تجربی و شاهد اختلاف دارد.

۳. . از دیدگاه مشتریها (گیرندگان مراقبت های دوران بارداری)، وضعیت کیفیت مشتری بعد از انجام مداخله بین گروه‌های تجربی و شاهد اختلاف دارد.

٤. گزارشات بیماران جهت سنجش کیفیت مراقبتهای ارائه شده به مادران باردار (کنترل وزنو فشار خون، بررسی ادم و واریس، آموزش های دوران بارداری، تجویز مکمل ها، ازمایشات دوران بارداری و....) با داده های ثبت شده در پرونده های خانوار آنها توافق آماری دارد.

**۸- اهداف طرح** (با توجه به مقدمه و بصورتی که قابل ارزیابی و اندازه گیری باشند، مشخص شود.)

الف) هدف کلی طرح ( اصولاً در برگیرنده کل عنوان طرح است)

**ارتقای کیفیت مراقبت های دوران بارداری از نگاه گیرندگان خدمت در شهر تبریز**

**ب) اهداف اختصاصی طرح:**

۱) اندازه گیری کیفیت خدمت (Service Quality) از نگاه گیرنده خدمت

۲) اندازه گیری کیفیت فنی(Technical Quality) از نگاه گیرنده خدمت

۳) اندازه گیری کیفیت فنی(Technical Quality) بر اساس اطلاعات موجود در پرونده گیرنده خدمت

٤) اندازه گیری کیفیت مشتری(Customer Quality) از نگاه گیرنده خدمت

٥) محاسبه شاخص کیفیت ( quality Index) از نگاه گیرنده خدمت

٦) مقایسه قابلیت تطابق جمع آوری داده ها "از نگاه گیرنده خدمت " با داده های جمع آوری شده از "پرونده خانوار"

۷) طراحی و اجرای مداخله بر اساس یافته های مراحل قبل

۸) اندازه گیری کیفیت خدمت (SQ) پس از انجام مداخله

۹) اندازه گیری کیفیت فنی (TQ ) پس از انجام مداخله

۱۰) اندازه گیری کیفیت مشتری (CQ) پس از انجام مداخله

۱۱) محاسبه شاخص کیفیت ( quality Index) پس از انجام مداخله

۱۲) مقایسه ابعاد کیفیت (SQ، TQ،CQ ) بعد از انجام مداخله بین گروه‌های تجربی و شاهد

**ج) اهداف کاربردی طرح**

۱) بررسی قابلیت و کارآئی مدل CQMH در اندازه گیری کیفیت خدمات ارائه شده به گیرنده گـان خـدمات پیشـگیری (بـه عنـوان مثـال: مادران باردار)

۲) بررسی اثر ارتقای دانش ومهارت مادران باردار در ارتقای کیفیت (کیفیت خدمت، کیفیت فنی و کیفیت مشتری) خدماتی که دریافت می کنند.

## ۹- نوع مطالعه:

**مطالعه از نوع کار آزمایی بالینی تصادفی کنترل دار خواهد بود.**

۱۰- کلیات روش اجرا : جمعیت هدف ، معیارهای ورود و خروج (Inclusion,Exclusion Criteria) ، توصیف دقیق گروههای مورد مطالعه ،حجم نمونه و روش نمونه گیری (Sampling)

با توجه به بند قبل موارد لازم برای هر نوع مطالعه را در این قسمت شرح دهید و در صورت نیاز می توانید از صفحات اضافی استفاده کنید ضمناً اگردر نوع مطالعه ،تدوین مورد خاصی ضرورت ندارد آن را ذکر کنید مثلاً اگر نیاز به تعیین حجم نمونه نیست ذکر شود که نیازی به تعیین حجم نمونه نیست تا مشخص شود که تمامی موارد دستور العمل لحاظ شده است .

در طرح تحقیقاتی حاضر کیفیت خدمات ارائه شده به مادران باردار با استفاده از مدل CQMH در سه بعد کیفیت خدمت، کیفیت فنی و کیفیت مشتری از نگاه گیرندگان خدمت سنجیده و ارتقا داده خواهد شد.

گروه هدف مادران باردار دارای پرونده درمراکز بهداشتی درمانی شهری شهر تبریز هستند که در آخرین ماه بارداری خود بوده و برای مراقبت دوران بارداری خویش تحت نظر مراکز بهداشتی درمانی بوده اند.

همچنین در بخش مداخله ای مطالعه گروه هدف مادران باردار دارای پرونده درمراکز بهداشتی درمانی شهری شهر تبریز هستند که در سه ماهه ی اول بارداری خود بوده و برای مراقبت دوران بارداری خویش تحت نظر مراکز بهداشتی درمانی بوده اند.

### معیار های ورود در طرح تحقیقاتی:

۱) باردار بودن مادر
۲) ساکن استان آذربایجان شرقی
۳) تحت مراقبت بودن در مراکز بهداشتی درمانی
٤) حداقل سه بار مراجعه به مرکز برای دریافت خدمات

### معیار های خروج از طرح تحقیقاتی:

۱) عدم توانائی لازم برای پاسخگوئی به سئوالات
۲) عدم تمایل به شرکت در طرح تحقیقاتی
۳) عدم شرکت در کلاس های آموزشی در مرحله مداخله طرح تحقیقاتی
٤) افراد مبتلا به بیماری شدید همراه

### حجم *نمونه و* روش نمونه گیری (Sampling):

**محاسبه حجم نمونه قبل از مداخله:** برای محاسبه حجم نمونه اطلاعات اولیه بر اساس مطالعه (پروژه کیفیت فنی مراقبـت هـای دوران بـارداری ) در قلب میانگین و انحراف معیار شاخص های SQ , CQ به دست آمد . به منظور تعیین حجم نمونه از فرمول

$$N = \frac{(z\ 1-\alpha/2z\ 1-\beta)^2 S^2}{d^2} = 97$$

استفاده شد که در آن $Z1\text{-}\alpha/2$ و $Z1\text{-}\beta$ به ازای $d=0.05$ و توان آزمون ۰,۸ به ترتیب برابر ۱,۹٦ و ۰,۸٤ می باشد. به علاوه S انحـراف معیـار و dحداکثر خطای قابل تحمل است که چون قرار است مداخله صورت گیرد میزانی از تغییر در نظر گرفته شد که می توان توسـط مداخلـه بـه آن دست یافت و برابر ۱/۲۰ مقدار میانگین در نظر گرفته شد. بر این اساس حجم نمونه برابر **۹٦,۸۲ (۹۷) در هر** یک از گروه های مـورد بررسـی محاسبه گردید.

نحوه ی نمونه گیری بدین صورت خواهد بود که ابتدا بر اساس نظر کارشناسان مرکز بهداشت شهرستان تبریز مراکز و پایگاه هـا را بـه سـه گـروه خوب، متوسط و ضعیف از نظر اجتماعی – اقتصادی طبقه بندی و سپس هر گروه را بصورت تصادفی به دو دسته تجربی و شاهد تقسیم و از هـر گروه ۱۰۰ نفر با استفاده از دفتر ثبت مراقبت زنان باردار در مراکز و پایگاه های بهداشتی لیست مـادران اسـتخراج و بطـور تصـادفی انتخـاب و بـا مادران باردار تماس گرفته می شود تا در صورت تمایل به شـرکت در مطالعـه مرکـز بهداشـتی درمـانی و یـا پایگـاه بهداشـتی مراجعـه نماینـد. و پرسشگران از طریق مصاحبه پرسش نامه را تکمیل خواهند کرد.

***ابزار گرد اوری داده ها:***

ابزار گرد آوری داده ها در این مطالعه پرسشنامه ای ۵ قسمتی خواهد بود که بخش اول قسمت اول مربوط به اسـتمرار دریافـت خـدمات از ارائـه دهنده گان مراقبت ها و ارائه دهنده گان خدمات و در بخش دوم کیفیت خدمت از دو جنبه عملکرد و اهمیت از دیدگاه گیرنده گان مـورد بررسـی قرار خواهد گرفت که این قسمت دارای ٤۰ سوال در قالب طیف لیکرتی اورده شده است.

قسمت دوم مربوط به کیفیت فنی مراقبت های دوران بارداری است که مراقبت های دوران بارداری در قالب "هیچ گاه" تا " هفت بـار" از شـرکت کننده گان پرسیده می شود. قسمت سوم پرسش نامه عینا شبیه قسمت دوم می باشد بااین تفاوت که از پرونده خـانوار مـادران بـاردار تکمیـل مـی گردد.

قسمت چهار مربوط به پرسشنامه کیفیت مشتری می باشد که دارای ۱٦ سوال در قالب طیف لیکرتی "موردی نداشتم"، "کاملامخالفم"، "مخـالفم"، "موافقم"، " کاملا موافقم" ارائه شده است.

قسمت نهایی مربوط به سوابق بارداری و همچنین اطلاعات فردی مادران باردار می باشد.

**۱۱- برای هر کدام از اهداف اختصاصی، اطلاعات زیر بصورت جداگانه تکمیل گردد:**

هدف اختصاصی **اول**: از شرکت کنندگان در مطالعه خواسته خواهد شد تا اهمیت هـر یـک از فاکتورهـای پرسشـنامه را در قالـب طیـف لیکرت چهار گزینه ای ۱۰ امتیازی در رنج مهم نیست =۰ ، نسبتا" مهم = ۳ ، مهم = ٦ و خیلی مهم = ۱۰ پاسخ دهد. عملکرد واقعـی سیسـتم ارائه کننده خدمت نیز در قالب طیف لیکرت چهار گزینه ای به شرح زیر امتیاز دهی خواهد شد. گزینه های "اغلب و همیشه" یـا " خـوب و عالی " = ۰ و گزینه های "هیچ وقت و گاهی اوقات" یا "ضعیف و متوسط"= ۱ . محاسبه کیفیت خدمت (SQ) و هر یک از ابعاد آن از نگاه ماردان باردار با استفاده از فرمول زیر:

$$\textit{Service Quality} = 10 - (\textit{Importance} \times \textit{Performance})$$

الف) خلاصه روش اجرا (توصیف دقیق گروههای مورد مطالعه ،تعداد نمونه هر گروه ،نحوه انجام تحقیق یا آزمایش)

بر اساس داده‌های حاصل از نمونه‌ی مورد بررسی از جامعه‌ی آماری، ابعاد کیفیت خدمت بر اساس آیتم‌های بدست آمده از بررسی متون به کمک پرسشنامه طراحی شده (که قبلا" روائی و پایائی آن بررسی شده است) اندازه گیری خواهد شد.

ب) جدول متغیرها (Variables):

| متغیر | کمی یا کیفی | نوع (مستقل – وابسته – مداخله گر) | روش کنترل و ارزیابی | تعریف علمی متغیر (در صورت نیاز) | مقیاس (scale) |
| --- | --- | --- | --- | --- | --- |
| اهمیت خدمت از نگاه مشتری | کیفی | مستقل | ۱۰ – ۰ | | رتبه ای |
| عملکرد واقعی سیستم | کیفی | مستقل | ۱ – ۰ | | اسمی |
| کیفیت خدمت | کمی | مستقل | ۱۰ – ۰ | | فاصله‌ای |
| سن | کمی | زمینه‌ای | | | نسبتی |
| جنس | کیفی | زمینه‌ای | | | اسمی |
| محل سکونت | کیفی | زمینه‌ای | | | اسمی |
| زمان تشخیص بارداری | کمی | زمینه‌ای | | | نسبتی |
| تحصیلات | کیفی | زمینه‌ای | | | رتبه ای |

## ج) روش تجزیه و تحلیل آماری داده ها ( روش آنالیز آماری مد نظر است نه صرفاً نرم افزار مورد استفاده )

به ازاء هر یک از آیتم های پرسشنامه فراوانی و درصد و برای نمرات مربوط به ابعاد کیفیت خدمت از میانگین و انحراف معیار ( و در صورت غیر نرمال بودن از میانه و IQR ) گزارش می شود همچنین برای بررسی ارتباط بین ابعاد کیفیت خدمت و فاکتور های دموگرافیک نسبتی از آزمون همبستگی پیرسون (در صورت غیر نرمال بودن از آزمون همبستگی اسپیرمن)، و با فاکتور های اسمی دو حالتی از آزمون T(و در صورت غیر نرمال بودن Mann-Whitney) و برای فاکتور های اسمی چند حالتی از آزمون ANOVA و همچنین برای بررسی ارتباط شاخص های کیفیت با متغییر های رتبه ای از آزمون همبستگی رتبه‌ای اسپیرمن استفاده خواهد شد. برای بررسی پایایی (همسانی درونی) پرسشنامه از شاخص α کرونباخ استفاده می شود. سطح معنی داری آزمون ها ۰٫۰۵ در نظر گرفته شده و برای انالیز داده ها از نرم افزار SPSS-19استفاده خواهد شد.

هدف اختصاصی **دوم، سوم :** اندازه گیری کیفیت فنی (TQ) از نگاه بیماران و اندازه گیری کیفیت فنی (TQ) از پرونده ماردان باردار

الف) خلاصه روش اجرا:

بر اساس شاخص های استاندارد مراقبت از مادران باردار (استانداردهای وزارت بهداشت درمان و آموزش پزشکی) پرسشنامه نهائی طرح تهیه و کیفیت فنی برای نمونه انتخاب شده اندازه گیری خواهد شد (که قبلا" روائی و پایائی پرسشنامه بررسی شده است). درا ین مطالعه کیفیت فنی بر اساس میزان تبعیت کارشناسان بهداشت خانواده مراکز بهداشتی درمانی از "استانداردهای مراقبت دوران بارداری" اندازه گیری می گردد (۱۸). برای این منظور سئوالات پرسشنامه توسط مصاحبه گرها از دو منبع کاملا" مستقل تکمیل خواهد شد؛ منبع اول: بیماران گیرندگان مراقبت های دوران بارداری و منبع دوم: پرونده خانوار مادران باردار که توسط کارشناسان بهداشت خانواده مراکز بهداشتی درمانی پس از مراقبت کامل مادران باردار تکمیل میشود.

ب) متغیرها

| متغیر | کمی یا کیفی | نوع (مستقل – وابسته – مداخله گر) | روش کنترل و ارزیابی | تعریف علمی متغیر (در صورت نیاز) | مقیاس (scale) |
|---|---|---|---|---|---|
| میزان تبعیت کارشناسان | کیفی | مستقل | | | رتبه ای |
| سن | کمی | زمینه‌ای | | | نسبتی |
| جنس | کیفی | زمینه‌ای | | | اسمی |
| محل سکونت | کیفی | زمینه‌ای | | | اسمی |
| زمان تشکیل پرونده | کمی | زمینه‌ای | | | نسبتی |
| تحصیلات | کیفی | زمینه‌ای | | | رتبه ای |

ج) روش تجزیه و تحلیل آماری داده ها ( روش آنالیز آماری مد نظر است نه صرفاً نرم افزار مورد استفاده )

به ازاء هر یک از آیتم های پرسشنامه فراوانی و درصد و برای نمرات مربوط به زیر شاخص و شاخص های کلی از میانگین و انحراف معیار ( و در صورت غیر نرمال بودن از میانه و IQR ) گزارش می شود همچنین برای بررسی ارتباط بین کیفیت فنی و فاکتور های دموگرافیک نسبتی از آزمون همبستگی پیرسون (در صورت غیر نرمال بودن از آزمون همبستگی اسپیرمن)، و با فاکتور های اسمی دو حالتی از آزمون T(و در صورت غیر نرمال بودن Mann-Whitney) و برای فاکتور های اسمی جند حالتی از آزمون ANOVA و همچنین برای بررسی ارتباط کیفیت فنی با متغییر های رتبه ای از آزمون همبستگی رتبه‌ای اسپیرمن استفاده خواهد شد. برای بررسی پایایی (همسانی درونی) پرسشنامه از شاخص α کرونباخ استفاده می شود. سطح معنی داری آزمون ها ۰٫۰۵ در نظر گرفته شده و برای انالیز داده ها از نرم افزار SPSS-19استفاده خواهد شد.

هدف اختصاصی **چهارم**: اندازه گیری کیفیت مشتری(CQ) از نگاه گیرندگان خدمت

الف) خلاصه روش اجرا:

کیفیت مشتری بر اساس پرسشنامه تغییر یافته Patient Activation Measure (PAM) (تهیه شده توسط پروفسور Hibbard و همکاران در دانشگاه اورگان آمریکا)، اندازه گیری خواهد شد (19). بر اساس تعریف کیفیت مشتری و سه ویژه گی آن بعصی از سئوالات تغییر یافته و سه سئوال به پرسشنامه ۱۳ سئوالی استاندارد اضافه شده است تا حدالامکان تمامی مفهوم کیفیت مشتری را پوشش دهد.

ب) متغیرها

| متغیر | کمی یا کیفی | نوع (مستقل – وابسته – مداخله گر) | روش کنترل و ارزیابی | تعریف علمی متغیر (در صورت نیاز) | مقیاس (scale) |
|---|---|---|---|---|---|
| کیفیت مشتری | کمی | وابسته | ۱۰۰ – ۰ | | فاصله‌ای |

ج) روش تجزیه و تحلیل آماری داده ها ( روش آنالیز آماری مد نظر است نه صرفاً نرم افزار مورد استفاده )

برای محاسبه نمره کیفیت مشتری نمرات خام بدست آمده ( rawcq ) به درجه بندی از ۱۰۰ تبدیل شد و متغیر actcq بدست خواهد آمد. به ازاء هر یک از آیتم های پرسشنامه فراوانی و درصد و برای نمرات مربوط به زیر شاخص و شاخص های کلی از میانگین و انحراف معیار ( و در صورت غیر نرمال بودن از میانه و IQR ) گزارش می شود. همچنین برای بررسی ارتباط بین کیفیت مشتری و فاکتور های دموگرافیک نسبتی از آزمون همبستگی پیرسون (در صورت غیر نرمال بودن از آزمون همبستگی اسپیرمن)، و با فاکتور های اسمی دو حالتی از آزمون T(و در صورت غیر نرمال بودن Mann-Whitney) و برای فاکتور های اسمی جند حالتی از آزمون ANOVA و همچنین برای بررسی ارتباط کیفیت مشتری با متغیر های رتبه ای از آزمون همبستگی رتبه‌ای اسپیرمن استفاده خواهد شد. برای بررسی پایایی (همسانی درونی) پرسشنامه از شاخص α کرونباخ استفاده می شود. سطح معنی داری آزمون ها ۰٫۰۵ در نظر گرفته شده و برای انالیز داده ها از نرم افزار SPSS-19استفاده خواهد شد.

هدف اختصاصی **پنجم**: محاسبه شاخص کیفیت ( Quality Index) از نگاه گیرنده خدمت

الف) خلاصه روش اجرا:

پس از جمع آوری داده های مربوط به جوانب مختلف کیفیت مراقبت های دوران بارداری شاخص کیفیت ( quality Index) از نگاه گیرنده خدمت که ترکیبی از نمرات مربوط به جوانب کیفیت است، محاسبه می شود.

ب) متغیرها

| متغیر | کمی یا کیفی | نوع (مستقل – وابسته – مداخله گر) | روش کنترل و ارزیابی | تعریف علمی متغیر (در صورت نیاز) | مقیاس (scale) |
|---|---|---|---|---|---|
| کیفیت خدمت | کمی | مستقل | ۱۰۰ – ۰ | | فاصله ای |
| کیفیت فنی | کمی | مستقل | ۱۰۰ – ۰ | | فاصله ای |
| کیفیت مشتری | کمی | مستقل | ۱۰۰ – ۰ | | فاصله ای |
| شاخص کیفیت | کمی | وابسته | ۱۰۰ – ۰ | | فاصله ای |

ج) روش تجزیه و تحلیل آماری داده ها ( روش آنالیز آماری مد نظر است نه صرفاً نرم افزار مورد استفاده )

برای محاسبه‌ی شاخص کیفیت  بر اساس نمرات حاصل از پرسشنامه‌ها مربوط به شاخص‌های CQ، TQ و SQ، با توجه به این که نمره‌ی مزبور به صورت ترکیبی از این سه شاخص است، برای محاسبه شاخص کیفیت (QI) ضرایب مربوط به این ترکیب از تحلیل آماری مولفه‌های اصلی (Principal Component Analysis - PCA) استفاده می‌شود. در این تحلیل امتیاز مربوط به کیفیت به صورت یک ترکیب خطی از نمرات سه شاخص فوق محاسبه می‌شود.  سطح معنی داری آزمون ها ۰،۰۵ در نظر گرفته شده و برای انالیز داده ها از نرم افزار SPSS-19 استفاده خواهد شد.

هدف اختصاصی **ششم:** مقایسه قابلیت توافق جمع آوری داده ها "از نگاه گیرنده خدمت " با داده های جمع آوری شده از "پرونده خانوار"

الف) خلاصه روش اجرا:

داده های مربوط به کیفیت فنی از دو منبع کاملا" مستقل و جدا از هم (مشتری خدمت و اطلاعات ثبت شده در پرونده گیرنده خدمت) جمع آوری شده و سپس میزان توافق بین این دو دسته از اطلاعات بررسی خواهد شد. وجود توافق بین این دو دسته از داده‌ها میزانی از دقت، صحت و قابلیت اعتماد دو شیوه جمع آوری داده را فراهم خواهد نمود.

ب) متغیرها

| متغیر | کمی یا کیفی | نوع (مستقل – وابسته – مداخله گر) | روش کنترل و ارزیابی | تعریف علمی متغیر (در صورت نیاز) | مقیاس (scale) |
|---|---|---|---|---|---|
| میزان تبعیت کارشناسان | کمی | | | | فاصله ای |
| دیدگاه گیرنده خدمت | کمی | | | | فاصله ای |

ج) روش تجزیه و تحلیل آماری داده ها ( روش آنالیز آماری مد نظر است نه صرفاً نرم افزار مورد استفاده )

برای ارزیابی تک تک آیتم ها از شاخص کاپای وزنی (Weighted kappa) و برای نمره کل از ICC (intraclass correlation coeficient ) و فاصله اطمینان ۹۵ درصدی آن استفاده خواهد شد. سطح معنی داری آزمون ها ۰،۰۵ در نظر گرفته شده و برای انالیز داده ها از نرم افزار SPSS-19 استفاده خواهد شد.

هدف اختصاصی **هفتم:** طراحی و اجرای مداخله بر اساس یافته های مراحل قبل

الف) خلاصه روش اجرا:

پس از انجام بررسی اولیه ۲۰ مرکز و پایگاه بهداشتی به تصادف انتخاب و پس از آن مراکز به دو گروه ۱) شاهد    ۲) مداخله: شامل آموزش دانش و مهارت  اختصاصی دوران بارداری، خود مراقبتی، آموزش فنون حل مسئله و ارتقای کیفیت در ابتدای بارداری و تشکیل پرونده تقسیم خواهند شد. مداخله طراحی شده برای گروه مداخله بعمل آمده و کیفیت خدمت، کیفیت فنی و کیفیت مشتری اندازه گیری خواهند شد. روش مداخله به این شکل خواهد بود که بعد از انجام مراحل ابتدایی مربوط به جمع آوری داده های مرتبط با جوانب مختلف کیفیت مراقبت های دوران بارداری نقاط ضعف و کاستی های آن ازدیدگاه مادران شناسایی و بر مبنای آن پمفلت و کتابچه آموزش توانمندی های لازم برای خود مراقبتی در طول بارداری و عوامل خطر مرتبط با بارداری و مراقبت های مورد نیاز برای کنترل این خطرات تهیه و در اختیار مادران گروه مداخله که تحت مراقبت های مراکز  و خانه های بهداشت بوده و در ماه های اولیه بارداری قرار دارند قرار خواهد گرفت. همچنین برای آموزش این توانمندی ها طی جلسه آموزشی برای هر فرد،آموزش های مورد نیاز برای تغییر و بهبود شیوه زندگی در مورد مسائل مرتبط با بارداری، آمادگی برای مقابله با خطرات دوران بارداری، افزایش توانمندی های مادر برای مراقبت و نگهداری از خود، جنین و نوزاد در طول دوران بارداری و بعد از زایمان

طراحی و ارائه خواهد شد. آموزش ها به صورت گروه های حمایتی متشکل از مادران باردار در گروه های ۱۰-۶ نفره به همراه کارشناسان بهداشت خانواده و مجریان طرح ساماندهی خواهد شد.

برای این منظور و با هدف طراحی مداخله حداقل ۵ جلسه بحث با صاحب نظران مربوطه برگزار و محتوای مداخله با مشورت و همفکری آنها و بر اساس مطالعات سایر کشور ها طراحی و تدوین خواهد شد.

هدف اختصاصی **هشتم، نهم، دهم و یازدهم**: اندازه گیری ابعاد کیفیت (SQ، TQ،CQ, ) و QI پس از انجام مداخله

الف) خلاصه روش اجرا:

بعد از انجام مداخله داده های مربوط به جوانب کیفیت دوباره از گروه های شاهد و مداخله (مادران باردار در ماه نهم بارداری) جمع آوری و همانند مرحله اول ابعاد کیفیت (SQ، TQ،CQ ) اندازه گیری و بر اساس این سه شاخص، شاخص کلی کیفیت (QI) محاسبه خواهد شد.

ب) متغیرها

| متغیر | کمی یا کیفی | نوع<br>(مستقل –وابسته – مداخله گر) | روش کنترل و ارزیابی | تعریف علمی متغیر<br>(در صورت نیاز) | مقیاس<br>(scale) |
|---|---|---|---|---|---|
| اهمیت خدمت از نگاه مشتری | کیفی | | ۱۰ – ۰ | | رتبه ای |
| عملکرد واقعی سیستم | کیفی | | ۱ – ۰ | | اسمی |
| کیفیت خدمت | کمی | | ۱۰ – ۰ | | فاصله ای |
| میزان تبعیت کارشناسان | کمی | | ۱۰۰ - ۰ | | فاصله ای |
| کیفیت مشتری | کمی | | ۱۰۰ – ۰ | | فاصله ای |
| شاخص کیفیت | کمی | | ۱۰۰ - ۰ | | فاصله ای |

ج) روش تجزیه و تحلیل آماری داده ها (روش آنالیز آماری مد نظر است نه صرفاً نرم افزار مورد استفاده )

به ازاء هر یک از آیتم های پرسشنامه فراوانی و درصد و برای نمرات مربوط به زیر شاخص و شاخص های کلی از میانگین و انحراف معیار ( و در صورت غیر نرمال بودن از میانه و IQR ) گزارش می شود همچنین برای بررسی ارتباط بین شاخص های کیفیت (QI) و فاکتور های دموگرافیک نسبتی از آزمون همبستگی پیرسون (در صورت غیر نرمال بودن از آزمون همبستگی اسپیرمن)، و با فاکتور های اسمی دو حالتی از آزمون T(و در صورت غیر نرمال بودن Mann-Whitney) و برای فاکتور های اسمی جند حالتی از آزمون ANOVA و همچنین برای بررسی ارتباط شاخص های کیفیت با متغییر های رتبه ای از آزمون همبستگی رتبه‌ای اسپیرمن استفاده خواهد شد. سطح معنی داری آزمون ها ۰٫۰۵ در نظر گرفته شده و برای انالیز داده ها از نرم افزار SPSS-19 استفاده خواهد شد.

هدف اختصاصی **دوازدهم**: مقایسه ابعاد کیفیت (SQ، TQ،CQ ) بعد از انجام مداخله بین گروه‌های مداخله و شاهد

الف) خلاصه روش اجرا:

پس از انجام مداخله طراحی شده برای گروه مداخله، مجددا" کیفیت خدمت، کیفیت فنی و کیفیت مشتری اندازه گیری شده و با نتایج مطالعه ی قبل از مداخله مقایسه خواهند شد.

ب) متغیرها

| متغیر | کمی یا کیفی | نوع (مستقل – وابسته – مداخله گر) | روش کنترل و ارزیابی | تعریف علمی متغیر (در صورت نیاز) | مقیاس (scale) |
|---|---|---|---|---|---|
| CQ در قبل | کمی | | | | فاصله ای |
| CQ در بعد | کمی | | | | فاصله ای |
| TQ در قبل | کمی | | | | فاصله ای |
| TQ در بعد | کمی | | | | فاصله ای |
| SQ در قبل | کمی | | | | فاصله ای |
| SQ در بعد | کمی | | | | فاصله ای |

ج) روش تجزیه و تحلیل آماری داده ها ( روش آنالیز آماری مد نظر است نه صرفاً نرم افزار مورد استفاده )

آزمون **Hoteling $T^2$** برای مقایسه همزمان شاخص های (SQوTQوCQ ) در دو گروه مداخله و شاهد مورد استفاده خواهد شد و برای مقایسه انفرادی هر یک از شاخص‌های فوق و شاخص QI از آزمون T مستقل استفاده خواهد شد و در صورت غیرنرمال بودن از آزمون ناپارامتری Mann-Whitney استفاده می شود. سطح معنی داری آزمون ها ۰,۰۵ در نظر گرفته شده و برای انالیز داده ها از نرم افزار **SPSS-19** استفاده خواهد شد.

**۱۲- ملاحظات اخلاقی** (در صورت نیاز فرم رضایتنامه آگاهانه ضمیمه گردد)

تمامی شرکت کنندگان در طرح پس از تکمیل و امضای فرم اعلام رضایت آگاهانه در طرح مشارکت داده خواهند شد. تمامی مراحل مطالعه شامل جمع آوری اطلاعات، ذخیره ، آنالیز و گزارش به صورت محرمانه و حفاظت شده بوده و کلیه اطلاعات فقط برای اعضای تیم تحقیق قابل دسترسی خواهد بود. تمامی پرسشنامه ها بدون ذکر نام تکمیل گردیده و نظرات شرکت کنندگان بدون اشاره به نام آنها در متن گزارش و مقالات خواهد آمد. روش مداخله به این شکل خواهد بود که بعد از انجام مراحل ابتدایی مربوط به جمع آوری داده های مرتبط با جوانب مختلف کیفیت مراقبت های دوران بارداری نقاط ضعف و کاستی های آن ازدیدگاه مادران شناسایی و بر مبنای آن پمفلت و کتابچه آموزش توانمندی های لازم برای خود مراقبتی در طول بارداری و عوامل خطر مرتبط با بارداری و مراقبت های مورد نیاز برای کنترل این خطرات تهیه و در اختیار مادران

گروه مداخله که تحت مراقبت های مراکز و خانه های بهداشت بوده و در ماه های اولیه بارداری قرار دارند قرار خواهد گرفت. همچنین برای آموزش این توانمندی ها طی ۳ جلسه آموزشی برای هر فرد،آموزش های مورد نیاز برای تغییر و بهبود شیوه زندگی در مورد مسائل مرتبط با بارداری ،آمادگی برای مقابله با خطرات دوران بارداری ،افزایش توانمندی های مادر برای مراقبت و نگهداری از خود ،جنین و نوزاد در طول دوران بارداری و بعد از زایمان طراحی و ارائه خواهد شد.آموزش ها به صورت گروه های حمایتی متشکل از مادران باردار در گروه های ۱۰-٦ نفره به همراه کارشناسان بهداشت خانواده و مجریان طرح ساماندهی خواهد شد.
تمامی مراحل انجام تحقیق و اهداف آن به مادران شرکت کننده در مطالعه توضیح داده خواهد شد و در صورت تمایل آنها برای شرکت در مطالعه از مادران باردار خواسته خواهد شد تا فرم رضایت آگاهانه را تکمیل و امضا نمایند. از آنجائیکه مداخله انجام گرفته فقط شامل آموزش حضوری و آموزش از طریق پمفلت و کتابچه خواهد بود، نه تنها هیچگونه آسیبی و یا صدمه ای برای مادران باردار نداشته بلکه باعث ارتقای دانش و مهارت مادران باردار شده و توانمندیهای آنها را در مشارکت در ارائه خدمات و اقدامات خود مراقبتی افزایش خواهد داد. مادران بار دار در گروه مداخله و یا شاهد در هر مرحله از تحقیق که هراده کنند میتوانند از پژوهش خارج شوند. این امر به هیچ وجه کیفیت خدمات دریافتی آنها را تحت تاثیر قرار نخواهد داد.

**۱۳- محدودیت ها و مشکلات احتمالی و راههای کاهش آنها :**

عدم مشارکت مادران باردار

عدم همکاری ارائه کنندگان خدمات

انصراف ماردان باردار از شرکت در مطالعه

## ۱٤- جدول زمانی مراحل اجرا طرح (GANTT CHART)

| ردیف | شرح هر یک از فعالیتهای اجرائی طرح به تفکیک | طول مدت (ماه) | زمان اجرا (ماه) | | | | | | | | | | | | | | | | | | | | | | |
|---|---|---|---|---|---|---|---|---|---|---|---|---|---|---|---|---|---|---|---|---|---|---|---|---|---|
| | | | ١ | ٢ | ٣ | ٤ | ٥ | ٦ | ٧ | ٨ | ٩ | ١٠ | ١١ | ١٢ | ١٣ | ١٤ | ١٥ | ١٦ | ١٧ | ١٨ | ١٩ | ٢٠ | ٢١ | ٢٢ | ٢٣ | ٢٤ |
| ١ | بررسی متون | ده ماه | ■ | ■ | ■ | | | | | | ■ | ■ | ■ | | | | | ■ | ■ | | | | | ■ | ■ | |
| ٢ | تهیه پرسشنامه | یک ماه | | | ■ | | | | | | | | | | | | | | | | | | | | | |
| ٣ | بررسی روایی و پایائی | یک ماه | | | | ■ | | | | | | | | | | | | | | | | | | | | |
| ٤ | جمع آوری داده- مرحله ۱ | دو ماه | | | | | ■ | ■ | | | | | | | | | | | | | | | | | | |
| ٥ | وارد کردن داده ها - ۱ | دو هفته | | | | | | | ■ | | | | | | | | | | | | | | | | | |
| ٦ | آنالیز داده ها - ۱ | یک ماه | | | | | | | ■ | | | | | | | | | | | | | | | | | |
| ٧ | نوشتن گزارش- ۱ | چهار ماه | | | | | | | | ■ | ■ | ■ | ■ | | | | | | | | | | | | | |
| ٨ | طراحی مداخله | دو ماه | | | | | | | | | | ■ | ■ | | | | | | | | | | | | | |
| ٩ | انجام مداخله | هفت ماه | | | | | | | | | | | | ■ | ■ | ■ | ■ | ■ | ■ | ■ | | | | | | |
| ١٠ | جمع آوری داده- مرحله۲ | دو ماه | | | | | | | | | | | | | | | | | | ■ | ■ | | | | | |
| ١١ | وارد کردن داده ها - ۲ | ۱۵ روز | | | | | | | | | | | | | | | | | | | ■ | | | | | |
| ١٢ | آنالیز داده ها -۲ | یک ماه | | | | | | | | | | | | | | | | | | | ■ | ■ | | | | |
| ١٣ | نوشتن گزارش نهائی | پنج ماه | | | | | | | | | | | | | | | | | | | | ■ | ■ | ■ | ■ | ■ |
| ١٤ | نوشتن مقاله | شش ماه | | | | | | | | | | ■ | ■ | ■ | | | | | | | | | | ■ | ■ | ■ |

**۱۵- منابع** (حداقل سه منبع اصلی مرتبط با موضوع ضمیمه شود)


1. Mainz J, Bartels P. Nationwide quality improvement. How are we doing and what can we do? *International Journal for Quality in Health Care.* January 24 2006;18:1-2.

2. Trento M, Passera P, Borgo E, et al. A 5-year randomised controlled study of learning, problem solving ability, and quality of life modification in people with Type 2 diabetes managed by group care. *Diabetes Care.* 2004;27:670-675.

3. Piette J, Weinberger M, Kraemer F, et al. Impact of automated calls with nurse follow-up on diabetes treatment outcomes in a department of veterans affairs health care system. *Diabetes Care.* 2001;24:202-208.

4. Donabedian A. *Explorations in quality assessment and monitoring. Volume 1: the definition of quality and approaches to its assessment*. Ann Arbor: Health Administration Press; 1980.

5. Kenagy J, Berwick D, Shore M. Service quality in health care. *The Journal of the American Medical Association.* 1999;281:661-665.

6. Tabrizi JS. *Quality of health care: the patients' perspective on quality of care for Type 2 diabetes* [Thesis for the degree of Doctor of Philosophy in Health Services Management]. Brisbane, School of Population Health, the University of Queensland; 12/06/2007.

7. WHO, departmant of making pregnancy safer, Strategic Approach to Improving Maternal and Newborn Survival and Health, Ensuring skilled care for every birth

8. WHO,implemention of the making pregnancy safer initiative(MPS)within the context of theroad mapfor accelerationg the attanimint of the millenium development goals (MDGs)reated to maternal and newborn health(MNH)in africa

9. Helton M: prenatal care. Woman health; 1997;34 (1):135-145

10. Petitte D, Hiatt R, Chin V, Groughan – minihane, M.An outcome evaluation of the content and quality of prenatal care. Birth 1991, 18: 21-25

11. - Stringer M. Issues in determining and measuring adequacy of prenatal care. Journal of Perinatology; 18: 68-73

12. WHO.Report of a technical working group, antenatal care. Geneva-

13. Siko SPL. Evaluations of quality of antenatal care at rural Health centers in Matebelend North Province Central. African Journal of Medicine 1996; 17: 423-34

14. Compos TP. "Infant Mortality in Rio Do Janeiro, Brazil: risk areas and distance Traveled by patient's to get to health care facilities. Review Paumam Salud Publication 2000; 8: 164-71

15. عسگری نژاد، معصومه ؛بخشی، حمید؛آگاهی ،نگرش و عملکرد زنان باردار رفسنجان نسبت به اهمیت مراقبت های دوران بارداری؛مجله علمی دانشگاه علوم پزشکی رفسنجان ،سال اول،جلد اول ،شماره ۳ ،۱۳۸۱

۱۶. دانش کجوری ،مهوش؛ کریمی، صدیقه؛ شکرآبی ،ربابه؛ حسینی، فاطمه: **بررسی رضایت زنان از مراقبت های دوران بارداری دریافت شده در مراجعین به خانه های بهداشت شهرستان شیروان چرداول؛** فصلنامه پرستاری دانشگاه علوم پزشکی ایران؛ دوره **هجدهم** ،پائیز،**شماره ۴۳**

۱۷. **میرمولایی،سیده طاهره؛خاکبازان،زهره؛کاظم نژاد،انوشیروان؛آذری، مهناز**:،میزان دریافت و رضایت از مراقبت های دوران بارداری، مجله دانشکده پرستاری و مامایی دانشگاه علوم پزشکی تهران (حیات) دوره ۱۳ ، شماره۲،۱۳۸٦، ۳۱-۴۰

18. Tabrizi JS, Wilson A, O'Rourke P and Coyne E. 2007. *Patient perspectives on consistency of medical care with recommended care in Type 2 diabetes.* Diabetes Care, 30 (11):2855-2856.

19. Hibbard JH, Mahoney E, Stockard J, et al. Development and testing of a short form of the patient activation measure. *Health Services Research.* 2005;40:1918-1931.

۱٦- آیا منبع دیگری ( به غیر از معاونت پژوهشی دانشگاه ) در تامین هزینه طرح مشارکت خواهد داشت ؟

بله □ خیر ■

لطفاً میزان مشارکت و چگونگی آن توضیح داده شود:

۱۷- آیا تمامی و یا قسمتی از طرح تحقیقاتی حاضر بعنوان پایاننامه دانشجویی می باشد ؟ بله ■ خیر□

در صورت مثبت بودن پاسخ :

مقطع تحصیلی دانشجو: کارشناسی ارشد رشته تحصیلی : مدیریت خدمات بهداشتی درمانی

دانشکده و دانشگاه محل تحصیل : بهداشت و تغذیه، دانشگاه علوم پزشکی تبریز

**۱۸- راهنمای تکمیل پرسشنامه را بدقت مطالعه، ضمن موافقت با آن، صحت مطالب مندرج در پرسشنامه را تأئید می نمایم.**

**نام و نام خانوادگی مجری طرح : دکتر جعفر صادق تبریزی**

**نام و نام خانوادگی مسئول سازمان یا محل اجرای طرح:**

دکتر احمد کوشا
معاون بهداشتی دانشگاه علوم پزشکی تبریز

**معاون پژوهشی**
**دانشگاه علوم پزشکی تبریز**
