# Supplementary material for: Effects of customer self-audit on the quality of maternity care in Tabriz: A cluster-randomized controlled trial
Source: PLoS One. 2018 Oct 11;13(10):e0203255. doi: 10.1371/journal.pone.0203255 (PMC6181295; doi:10.1371/journal.pone.0203255)
Supplement: S6 File — (PDF) [file pone.0203255.s006.pdf]

Research Deputy

Research Proposal

Name and surname: Dr.Jafar-Sadegh Tabrizi

Title: Improving quality of maternity care from the perspective of pregnant women in tabriz.

If the final result of this project result in publication of an article, to declare the source of financing (Department of Tabriz University of Medical Sciences) is necessary.

Deputy of Research and Technology, 2th Floor, #2 Central Building, Tabriz, IranPhone: +98(41) 33357310

1- Title:

Title in Persian:

ارتقای کیفیت مراقبت های دوران بارداری از نگاه مادران باردار در شهر تبریز

Title in English:

Improving quality of maternity care from the perspective of pregnant women in tabriz.

2- The present research project is in line with which one of the university research priorities?

A-Health, Prevention and Lifestyle:

8- Maternal care and high risk pregnancies

3- Information on Executive and Project Location:

Name and surname	Jafar-Sadegh Tabrizi.
Address and telephone work	School of Health and Nutrition, Department of Management and Public Health.
Job and current organizational post	Faculty member.
Scientific degrees and academic discipline	PhD in Health Services Management.
Location (s) of the project	Tabriz health centers and health posts.
Account number / National Bank branch University	
Cellular phone	
tabrizijs@tbzmed.ac.ir	tabrizijs@tbzmed.ac.ir

4- Individual information:

Name and surname	Job	Scientific degrees and academic discipline	Type of Collaboration*	Total hourshave spent for reserach	Fee per hour	Total (Rls)	sign
**Executive and core collaborators**							
Jafar-Sadegh Tabrizi.	Faculty member.	PhD in Health Services Management.	Outline the proposal, otline the questionnaire, design the intervention, before-after data Analysis, writing the report and article.	500			
Mostafa Farahbakhsh	IT Director of Provincial Health Center	Physician, par faculty	Outline the proposal, otline the questionnaire, design the intervention, writing the report and article.	350			
Dr.Mardi		Physician	design the intervention, Data collection	100			
Mojtaba Mohammadzade		Physician	design the intervention, before-after data collection, writing the report and article.	250			
Mrs Afsharnia	Tabriz Health Center's Family Health Expert	Health Expert	design the intervention, educating the Family Health Expert, support groups education, Data collection	200			
Mrs Farshbaf	East-Azerbayjan Provincial Health Center's Family Health Expert	Health Expert	design the intervention, educating the Family Health Expert, support groups education, Data collection。	200			
Mohammad Asghari	Faculty member.	PhD in of Biostatistics	Statistical consultation on proposals, collaboration in intervention data analysis.	100			
Kamal Gholipour	student	MSc of Health Services Management	Outline the proposal, otline the questionnaire, design the intervention, before-after data Analysis, writing the report and article.	400			
Shabnam Iezadi	student	MSc of Health Services Management	Outline the proposal, otline the questionnaire, design the intervention, before-after data Analysis, writing the report and article.	200			
Rahime Alipour	student	BSc of Health Services Management	Data collection, data Analysis, writing the report and article.	200			
**others**							
Health center employee	Tabriz health center's staff	Public health expert	Prenatal care delivery, Education, Data collection	1000			
Total cost(RLs)				Type of cooperation in relation toimplementation of the project, should be mentioned for each individual accurately and separately.			

5- Introduction and statement of the problem:

In today's world, especially in developing countries, limited resources (financial, human, equipment, etc.) as well as the emergence of new technologies on the one hand and increasing the awareness, knowledge and expectations of health care recipients to receive high-quality services on the other hand havecaused continuous quality improvement initiatives to be considered essential for the life of health system.Improvement of

servicequality not only results in providing optimum services to clients (health care recipients), reducing the time taken to get services and reducing costs for providing services, but also dramatically increases the satisfaction and willingness of clients to refer again.The above evidence suggests the fact that improving the quality of services provided at health units should be one of the basic fundamental objectives and priorities of health systems and managers.Due to these conditions, managers need to improve the quality of their work as a priority and make it a popular culture for all employees at all levels of their organization to participate. .In addition to determine the scope of their activities, customers and providers, all employees working in each unit sholud have a regular quality improvementschedule based on priority areas of their work.Obviously, customer participation, as effective member of the health team, is of the utmost importance to improve the quality of services and requires special attention of all managers and employees(1-3).

Given the importance of improving the quality of health care, method of measuring the quality of services and comprehensive measurement is extremelyimportant.Todays, different approaches and models are used to measure the quality of services. For example, in 1980 Dvnabdyn (4), a pioneer in health care quality improvement provided a model which isincluding: 1) technical quality, 2) structure quality and 3) process quality for measuring the quality of services, and researchers around the world used it for a long time.This model was changed and completed in 1999 by Kenagy and Berwick and was introduced as an effective model for measuring the quality of health care(5).The model have been used in several research by different researchers since the creation.

Based on the definitions by Kenagy and Berwick,Technical Quality (TQ) is what the customer receives versus what is presented as a standard serviceon the basis of scientific evidence.This dimension of the quality is often a reflection of the knowledge, skills and

capabilities of service providers.Unlike TQ, Service Quality(SQ) is related to terms and conditions in whichclients recieve services, and almost indicates the meathods of providing care and the setting in which services are delivered(5).

Since both above-mentionedmodels , measure the quality of servicesbased on clinical(TQ) and non-clinical (SQ) dimensions, it seems that the other crucial dimension in the provision of health services has been neglected. This dimension, namely Customer Quality (CQ), is related to health care recipients and refers tothe knowledge of clientsabout services they receive, clients' features and capabilities that undoubtedly has important effect on the manner ofcaredelivery. Clients' capabilities are resulted in more participation of them in care, involvement in key decisions, better service delivery process, intime implementation of intervention in cases of necessity and constructive engagement with service providers. Due to the necessity of the above points, at this research proposal, Comperhensive Quality Measurment in Healthcare (CQMH) model which was presented by Tabrizi et al. in 2007, encompasses all three dimensions of quality including: TQ, SQ and CQ, and will be used to comprehensively assess the quality. CQ is a set of features and capabilities required by the service recipients in order to effectively participate in the process of health care, key decision making and an accurate and timely intervention (6).

Because the health of pregnant women is one of the most important and the most basic health needs of each community,the current research project will study the applicability of CQMH model for measuring quality of care provided for pregnant women during the nine-month pregnancy . However, each year about 99% of the 529,000 maternal deaths is related to pregnancy and childbirth, and 98% of 5.7 million deaths occur in developing countries(7).It is estimated that, in developing countries, women are 140 times more susceptible to the pregnancy related risks. In the Millennium Development Goals (Goal 3, 4 and 5) women's health and welfare are concidered as the main development indicators (8). Pregnant women's health encompasses different aspects, and different components are essentioal in achieving women's health goals, including: access to trained health workers, social awareness about the need for healthy pregnancy, improving the health of women, improving the performance of health care providers, the maternal mortality monitoring system, guiding and organizing performance of traditional birth attendants, increasing the efficiency of existing facilities and the development of strategic policies for improving the quality of prenatal care, childbirth, family planning and obstetric facilities.

Prenatal care refers to a set of services that systematically monitors mother's health status during pregnancy, and prepares the conditions for a healthy baby birth.Prenatal care is one of the most valuable and most economical health plans, which shows relatively good effects on mother-baby health status and reduces health system costs(9).Most studies have determined adequacy of prenatal care based on the duration and the number of visits so they didn'tpay attention to the content of services. While, the content of a prenatal care visits is important as much as the duration and the frequency of visits (10, 11). Results of studies conducted in developed countries indicated that high quality prenatal care as an effective intervention leads to the reduction in Infant Mortality Rate-IMR, Maternal MortalityRate-MMR and Perinatal Mortality Rate-PMR, so, paying attention to prenatal care, monitoring, evaluatimg and improving quality of such care and having responsibility to meet pregnant women's needs and expectations become more important(12, 13 and 14).

Some research has shown a close relationship between the quality of prenatal care and knowledge and attitude of recipients of care.So that, the more knowledge and information clients have, the higher quality of care they receive. Studies also show that mothers' satisfaction with the quality of information provided for them about the prenatal care is low, and this is one of the problems in achieving objectives of prenatal care and quality improvement (15, 16 and 17).

6- Definition of specific words

Service Quality: service quality(SQ) is a dimension of quality related to terms and conditions in which clients recieve services, and almost indicates the meathods of providing care and the setting in which services are delivered (6).

Technical Quality: technical quality (TQ) is the clinical and technical dimension of quality and defined as what customer receives versus what is presented as a standard service on the basis of scientific evidence (5).

Customer Quality: CQ is a new dimension of quality and defined as a set of features and capabilities required by service recipients in order to effectively participate in the process of health care, key decision making and accurate and timely intervention(6).

7- Research hypotheses:

A- From the perspective of clients (prenatal care reciepents), the TQ scores are different between experimental and control groups after the intervention.

B- From the perspective of clients (prenatal care reciepents), the SQ scores are different between experimental and control groups after the intervention.

C- From the perspective of clients (prenatal care reciepents), the CQ scores are different between experimental and control groups after the intervention.

D- Customer reporteddata for measuring the quality of care provided for pregnant women (weight and blood pressure control, edema and varicose examination, prenatal training, supplementation, routine tests during pregnancy, etc.) arein agreement with the medical recorded data.

8- Research objectives:

- Goal (Basically, includes the title of research)

Improving the quality of prenatal care, from the perspective of service recipients in Tabriz.

- Research objectives:

1- Measuring the Service Quality from the perspective of the customers.
2- Measuring the Technical Quality from the perspective of the customers.
3- Measuring the Technical Quality based on medical recorded data .
4- Measuring the Customer Quality from the perspective of the customers.
5- Calculating the Quality Index from the perspective of the customers.
6- Comparing the agreement of between customer reported and medical recorded data.
7- Designing and implementing interventions based on the findings of previous steps.
8- Measuring Service Quality after the intervention.
9- Measuring Technical Quality after the intervention.
10- Measuring Customer Quality after the intervention.
11- Calculating the Quality Index after intervention.
12- Comparing dimensions of quality (CQ, TQ, SQ) between experimental and control groups after the intervention.

9- Study design:

Randomized controlled clinical trials

10- Sampling:

The study sample was calculated using G*Power software. Based on 80% power and a confidence level of 95%, and considering 0.05 as maximum tolerable error rate (d) and based on 0.38 standard deviation of adherence rate in a previous study we estimated a sample size of 97 in each group as: $n = ((Z\ 1\text{-}\alpha^2 / Z\ 1 - \beta)^2\ s^2) / d^2$

Based on the Tabriz district health center experts, health center and health post will be classified into three subgroup as high, medium and low socioeconomic situation and in each subgroup 3-4 health centers and health posts randomly will be selected as intervention and control group. Then 100 participants randomly will be selected from the lists of pregnant women registered at health centers and health posts. Pregnant women will be contacted in case they want to participate in the study referred to health centers or health posts.

In this research project quality of delivered care for pregnant women will be assessed and improved based on CQMH in three dimension as; service, technical and customer quality from the perspective of pregnant women.

Eligible participants in descriptive phase will be pregnant women at near term (nine-month) stage who had re-ceived at least three episodes of pregnancy care and lived in Tabriz.

Eligible participants in intervention phase will be pregnant women registered at health centers and health posts in their first trimester of pregnancy.

Inclusion Criteria:

- Being pregnant
- pregnant women who lived in Tabriz
- pregnant women who had re-ceived pregnancy care from health center and health post
- pregnant women who had re-ceived at least three episodes of pregnancy care

Exclusion Criteria:

- lack of ability to answer the questions
- unwillingness to participate in the research project
- unwillingness to participate in training courses in the intervention research project
- patients with severe disease

11- give enough information about each objective.

Objective No 1: Measuring the Service Quality from the perspective of the customers.

B) Materials and Methods Summary:

participants will be asked to evaluate the performance of that aspect according to either their understanding of the quality of care or the real experience they gained when they received care that pertained to that aspect over the past year. Importance of SQ was scored on a four-point Likert scale that ranged from 0 to 10 as: 0= not important, 3= may be important, 6=

important and 10= very important. Perceived performance of care received was valued by a four-level Likert scale with the following categories: ‘poor, fair, good and excellent’. This scale was dichotomized as: 0= good/excellent’ and 1= poor/fair. Each aspect of SQ of prenatal care will be calculated according to the following formula:

SQ = 10 – (Importance × performance).

B) Variables:

Variable	Qualitative/ Quantitative	Dependent/independent/ demographics	scale
Importance	Qualitative	Dependent	Ordinal
Performance	Qualitative	Dependent	Ordinal
SQ	Quantitative	Dependent	Interval
Age	Quantitative	demographics	Ratio
Sex	Qualitative	demographics	Nominal
Place of residence	Qualitative	demographics	Nominal
Awareness of pregnancy	Quantitative	demographics	Ratio
Education	Qualitative	demographics	Ordinal

C) Statistical analysis

Frequencies and percentages extracted from the obtained data will be used to describe the demographic information of participants. The obtained means (standard deviations), (median (IQR) for abnormal data) will be used to report SQ scores. Pearson correlation (Spearman correlation coefficient for abnormal data) and Independent Samples Test (Mann-Whitney for abnormal data), ANOVA, and Tukey HSD Post Hoc Test will be used to compare SQ score and its aspects between numerical and categorical variables, respectively. The instrument reliability will be confirmed using Cronbach's alpha index, according to a pilot study on a group of 30 patients. P values ≤ 0.05 will be considered as statistically significant. The data will be analyzed using the SPSS-19 statistical software.

Objective No 2 and 3: Measuring the Technical Quality from the perspective of the customers.

B) Materials and Methods Summary:

Study questionaire will be developed based on the protocols of the Iranian Ministry of Health (MOH) for pregnancy care and TQ will be assessed using this instrument.The technical quality will be measured based on compliance with "Prenatal care standards" in health

centers by family health experts (18). For this purpose the questionnaire will be completed by interviewer, from two independent sources; the first source is pregnant women and second source is medical and household record of pregnant women were completed by health professionals in health centers after completing the care of pregnant women.

B) Variables:

Variable	Qualitative/ Quantitative	Dependent/independent/ demographics	scale
Adherence rate	Qualitative	Dependent	Ordinal
Age	Quantitative	demographics	Ratio
Sex	Qualitative	demographics	Nominal
Place of residence	Qualitative	demographics	Nominal
Registration time	Quantitative	demographics	Ratio
Education	Qualitative	demographics	Ordinal

C) Statistical analysis

Frequencies and percentages extracted from the obtained data will be used to describe the demographic information of participants. The obtained means (standard deviations), (median (IQR) for abnormal data) will be used to report TQ scores. Pearson correlation (Spearman correlation coefficient for abnormal data) and Independent Samples Test (Mann-Whitney for abnormal data), ANOVA, and Tukey HSD Post Hoc Test will be used to compare TQ score between numerical and categorical variables, respectively. The instrument reliability will be confirmed using Cronbach's alpha index, according to a pilot study on a group of 30 patients. P values ≤ 0.05 will be considered as statistically significant. The data will be analyzed using the SPSS-19 statistical software.

Objective No 4: Measuring the Customer Quality from the perspective of the customers.

B) Materials and Methods Summary:

CQ will be assessed using modified Patient Activation Measure quastionaire (PAM) were developed by Hibbard et al in oregan university (19). Based on CQ defination 6 qustions were added to 13 items PAM quastionaire.

B) Variables:

Variable	Qualitative/ Quantitative	Dependent/independent/ demographics	scale
CQ	Quantitative	Dependent	Ordinal

C) Statistical analysis

Raw scores will be normalized to zero-100, to compute the active CQ scores. Frequencies and percentages extracted from the obtained data will be used to describe the demographic information of participants. The obtained means (standard deviations), (median (IQR) for abnormal data) will be used to report CQ scores. Pearson correlation (Spearman correlation coefficient for abnormal data) and Independent Samples Test (Mann-Whitney for abnormal data), ANOVA, and Tukey HSD Post Hoc Test will be used to compare CQ score between numerical and categorical variables, respectively. The instrument reliability will be confirmed using Cronbach's alpha index, according to a pilot study on a group of 30 patients. P values ≤ 0.05 will be considered as statistically significant. The data will be analyzed using the SPSS-19 statistical software.

Objective No 5: Calculating the Quality Index from the perspective of the customers.

B) Materials and Methods Summary:

After collecting data on various dimensions of prenatal care quality, quality Index (QI) from the perspective of participants will be calculated using as combination of quality dimensions scores (SQ, CQ, TQ).

B) Variables:

Variable	Qualitative/ Quantitative	Dependent/independent/ demographics	scale
SQ	Quantitative	independent	Interval
TQ	Quantitative	independent	Interval
CQ	Quantitative	independent	Interval
Quality Index	Quantitative	Dependent	Interval

C) Statistical analysis

For calculating quality index based on SQ, CQ and TQ scores and considering this fact QI calculated by combining SQ, CQ and TQ scores, Principal Component Analysis (PCA) will be used to indicate The coefficient of each dimensions. In this analysis, the QI will be calculated as a linear combination of the scores of the three dimensions. P values ≤ 0.05 will be considered as statistically significant. The data will be analyzed using the SPSS-19 statistical software.

Objective No 6: Comparing the agreement of between customer reported and medical recorded data.

B) Materials and Methods Summary:

Data relate to TQ will be completed from two independent sources; the first source is pregnant women and second source is medical and household record of pregnant women and then agreement between two source of data will be assessed. The presence of agreement between two source of data indicates the precision, accuracy and reliability of these two source of data.

B) Variables:

Variable	Qualitative/ Quantitative	Dependent/independent/ demographics	scale
Adherence rate based on medical record	Quantitative	independent	Interval
Customer reported data	Quantitative	independent	Interval

C) Statistical analysis

Kappa Weighted will be used to assess the agreement between each item and ICC (interclass correlation coefficient) will be used to assess the agreement in total TQ score. P values ≤ 0.05 will be considered as statistically significant. The data will be analyzed using the SPSS-19 statistical software.

Objective No 7: Designing and implementing interventions based on the findings of previous steps.

B) Materials and Methods Summary:

After descriptive phase 20 health center and health post randomly will be selected and allocated as intervention and control group. Participants in intervention group not only received national guideline-based care in individual visits, but also they took part in self-care activities and attended support group meetings and will be recived educational tips about experiential learning, coping, problem-solving and goal-setting were given to them by a family health expert, a midwife and a doctor in each session. After conducting intervention SQ, TQ and CQ scores will be calculated in both intervention and control groups. Intervention design will be developed after the conduction of initial and descriptive stages of study weaknesses and

shortcomings from the perspective of pregnant women will be identified and based on these findings capability building booklet for self-care activities and controlling risk factors during pregnancy were developed and will be provided to women in the intervention group in their first trimester of pregnancy. Educational session will be conducted to build Capabilities

regard to change management, life style, readiness to manage the risk factor during pregnancy, increased capabilities for care and maintenance of fetus and infant during pregnancy and after delivery. Also 6-10 participants in intervention group willbe grouped based on their gestational age and will be attendant in support group sessions during pregnancy by a family health expert, a midwife and a doctor in each session. For this purpose and to develop interventin package 5 focus group will be conducted by informants and intervention conent will be developed using their comments and international experiences.

Objective No 8, 9, 10 and 11: Measuring SQ, TQ, CQ and QI after the intervention.

B) Materials and Methods Summary:

After conducting intervention data regard to SQ, TQ and CQ in both intervention and control groups will be completed and SQ, TQ and CQ scores will be calculated and QI will be calculated as a linear combination of the scores of the three dimensions.

B) Variables:

Variable	Qualitative/ Quantitative	Dependent/independent/ demographics	scale
Importance	Qualitative	Dependent	Ordinal
Performance	Qualitative	Dependent	Nominal
SQ	Quantitative	independent	Interval
Adherence rate based on medical record (TQ)	Quantitative	independent	Interval
CQ	Quantitative	independent	Interval
Quality Index	Quantitative	Dependent	Interval

C) Statistical analysis

Frequencies and percentages extracted from the obtained data will be used to describe the demographic information of participants. The obtained means (standard deviations), (median (IQR) for abnormal data) will be used to report SQ, TQ, CQ and QI scores. Pearson correlation (Spearman correlation coefficient for abnormal data) and Independent Samples Test (Mann-Whitney for abnormal data), ANOVA, and Tukey HSD Post Hoc Test will be

used to compare SQ, TQ, CQ and QI score between numerical and categorical variables, respectively. P values ≤ 0.05 will be considered as statistically significant. The data will be analyzed using the SPSS-19 statistical software.

Objective No 12: Comparing dimensions of quality (CQ, TQ, SQ) between experimental and control groups after the intervention.

B) Materials and Methods Summary:

The SQ, TQ and CQ scores after intervention will be compared with the result of descriptive phase of study.

B) Variables:

Variable	Qualitative/ Quantitative	Dependent/independent/ demographics	scale
CQ in case group	Quantitative	Dependent	Interval
CQ in control group	Quantitative	Dependent	Interval
TQ in case group	Quantitative	Dependent	Interval
TQ in control group	Quantitative	Dependent	Interval
SQ in case group	Quantitative	Dependent	Interval
SQ in control group	Quantitative	Dependent	Interval

C) Statistical analysis

Hoteling T^2 will be conducted to simultaneous compare of SQ, TQ and CQ between intervention and control group also Independent Samples Test (Mann-Whitney for abnormal data) will be used to compare SQ, TQ, CQ and QI score between intervention and control group individualty. P values ≤ 0.05 will be considered as statistically significant. The data will be analyzed using the SPSS-19 statistical software.

12- Ethical Consideration:

All the participants after completing and signing the informed consent form will be participated in study. All stage of study contains data collection, storage, analysis and reports will be conducted confidential and all information will be accessible only to members of the research team. All questionnaires will be completed anonymously and opinions of the participants will be reported as reports and articles without mentioning their names.

Intervention design will be developed after the conduction of initial and descriptive stages of study weaknesses and shortcomings from the perspective of pregnant women will be identified and based on these findings capability building booklet for self-care activities and controlling risk factors during pregnancy were developed and will be provided to women in the intervention group in their first trimester of pregnancy. Educational session will be

conducted to build Capabilities regard to change management, life style, readiness to manage the risk factor during pregnancy, increased capabilities for care and maintenance of fetus and infant during pregnancy and after delivery. Educational session will be conducted to build Capabilities regard to change management, life style, readiness to manage the risk factor during pregnancy, increased capabilities for care and maintenance of fetus and infant during pregnancy and after delivery. Also 6-10 participants in intervention group will be grouped based on their gestational age and will be attendant in 5 support group sessions during pregnancy by a family health expert, a midwife and a doctor in each session. For this purpose and to develop interventin package 5 focus group will be conducted by informants and intervention conent will be developed using their comments and international experiences.

All stages of the research and its objectives will be explained to mothers participating in the study. So, they be asked to complete and sign an informed consent form. Since the intervention only consisted of training and education through pamphlets and handbooks, it will be expected no harm and side effect for participants. But also it will be expected knowledge and skills of pregnant women regard to self-care and maternity services will be imroved.

Pregnant women in the intervention or control groups in any phase of the research that they desire, can be excluded from research. This does not affect the quality of the services they will be received.

13- Potential Limitation:

- The lack of participation of pregnant women
- Lack of cooperation from service providers
- Last to flow-up

14- GANTT CHART

	**Activity**	**Duration (Month)**	**Time (Month)**																							
			1	2	3	4	5	6	7	8	9	10	11	12	13	14	15	16	17	18	19	20	21	22	23	24
	Literature review	10	■	■	■						■	■	■					■	■					■	■	
	Questionnaire development	1			■																					
	Validity and Reliability	1				■																				
	Data collection-1	2					■	■																		
	Data entry - 1	1							■																	
	Data analysis - 1	1							■																	
	Report development - 1	4								■	■	■	■													
	Intervention development	2										■	■													
	Intervention	7												■	■	■	■	■	■	■						
	Data collection-2	2																		■	■					
	Data entry - 2	1																			■					
	Data analysis - 2	1																			■	■				
	Report development - 2	5																				■	■	■	■	■
	Manuscript developmen	6										■	■	■										■	■	■

15- References:

1. Mainz J, Bartels P. Nationwide quality improvement. How are we doing and what can we do? International Journal for Quality in Health Care. January 24 2006; 18: 1-2.
2. Trento M, Passera P, Borgo E, et al. A 5-year randomized controlled study of learning, problem solving ability, and quality of life modification in people with Type 2 diabetes managed by group care. Diabetes Care. 2004; 27: 670-675.
3. Piette J, Weinberger M, Kraemer F, et al. Impact of automated calls with nurse follow-up on diabetes treatment outcomes in a department of veterans' affairs health care system. Diabetes Care. 2001; 24: 202-208.
4. Donabedian A. Explorations in quality assessment and monitoring. Volume 1: the definition of quality and approaches to its assessment. Ann Arbor: Health Administration Press; 1980.
5. Kenagy J, Berwick D, Shore M. Service quality in health care. The Journal of the American Medical Association. 1999; 281: 661-665.
6. Tabrizi JS. Quality of health care: the patients' perspective on quality of care for Type 2 diabetes [Thesis for the degree of Doctor of Philosophy in Health Services Management]. Brisbane, School of Population Health, the University of Queensland; 12/06/2007.
7. WHO, department of making pregnancy safer, Strategic Approach to Improving Maternal and Newborn Survival and Health, Ensuring skilled care for every birth
8. WHO, implementation of the making pregnancy safer initiative (MPS) within the context of the road map for accelerating the attainment of the millennium development goals (MDGs) reated to maternal and newborn health (MNH) in Africa
9. Helton M: prenatal care. Woman health; 1997; 34 (1):135-145
10. Petitte D, Hiatt R, Chin V, Groughan – minihane, M.An outcome evaluation of the content and quality of prenatal care. Birth 1991, 18: 21-25

11. - Stringer M. Issues in determining and measuring adequacy of prenatal care. Journal of Perinatology; 18: 68-73
12. WHO. Report of a technical working group, antenatal care. Geneva-
13. Siko SPL. Evaluations of quality of antenatal care at rural Health centers in Matebelend North Province Central. African Journal of Medicine 1996; 17: 423-34
14. Compos TP. "Infant Mortality in Rio Do Janeiro, Brazil: risk areas and distance Traveled by patient's to get to health care facilities. Review Paumam Salud Publication 2000; 8: 164-71
15. HajiKazemi E, SH. Hossein Mohseni, Oskouie F, H. Haghani. The association between knowledge, attitude and performance in pregnant women toward dental hygiene during pregnancy. IJN. 2005; 18 (43):31-38
16. M. Danesh Kojuri, S. Karimi, R. Shekarabi, F. Hossinei. A study of satisfaction with prenatal care services in the women attending to the health centers of the Shirevan Chardavel (Iran), in 2005. IJN. 2005; 18 (43):61-69
17. Mirmolaei S, Khakbazan Z, Kazemnejad A, Azari M. Prenatal Care Utilization Rate and Patients Satisfaction. Hayat. 2007; 13 (2):31-40
18. Tabrizi JS, Wilson A, O'Rourke P and Coyne E. 2007. Patient perspectives on consistency of medical care with recommended care in Type 2 diabetes. Diabetes Care, 30 (11):2855-2856.
19. Hibbard JH, Mahoney E, Stockard J, et al. Development and testing of a short form of the patient activation measure. Health Services Research. 2005; 40: 1918-1931.

16. Does another source (other than the Research Deputy) will participate in financing the project?

Yes ☐ No ■

Please rate of participation and how it is explained:

17. Is all or part of the research project as a thesis student? Yes ■ No☐

if the answer is yes:

Student Degree: MSc in Health services management

College and university: faculty of management and medical informatics, Tabriz University of Medical Sciences

18. Carefully administered proposal, agreed with it, I can confirm the accuracy of the contents of the questionnaire.

Project Executor: Dr.Jafar-sadegh Tabrizi

The name of the organization or the location of the project head:

Dr.Ahmad Koosha
Health deputy Tabriz University of Medical Sciences

Research Deputy
Tabriz University of Medical Sciences
