# Supplementary material for: Effects of customer self-audit on the quality of maternity care in Tabriz: A cluster-randomized controlled trial
Source: PLoS One. 2018 Oct 11;13(10):e0203255. doi: 10.1371/journal.pone.0203255 (PMC6181295; doi:10.1371/journal.pone.0203255)
Supplement: S7 File — (PDF) [file pone.0203255.s007.pdf]

Service Quality measure

INSTRUCT IONS:

In this section of the questionnaire we are interested to know what you think about the quality of non-medical aspects of diabetes care that you actually experience. Please read the example carefully and answer all questions.

Example: ■ Having the right to see a specialist, if you wish to:

Never 1 ❑. Sometimes 2■ Usually 3 ❑ Always 4 ❑

If you mark the second box in the example, it means that you have thought about that for a moment and you think you had just a few chances to visit a specialist who you specifically wanted to see, so you marked "Sometimes".

A: Choice of provider

1 To **what type of medical service** do you go for your usual maternity care needs? (you can mark more than one)

General practitioner (GP)	1 ❑
gynecologist	2 ❑
Other specialists	3 ❑
Midwife or health worker	4 ❑
Nurse practitioner	5 ❑
No defined principal provider	6 ❑

2 have you met one particular health care provider in your pregnancy period?

Yes 1 ❑ No 2❑

3 have you met one particular gynecologist in your pregnancy period?

Yes 1 ❑ No 2❑

importance						**performance**			
**Never**	**Sometimes**	**Usually**	**Always**			**Never**	**Sometimes**	**Usually**	**Always**
1 ❑	2 ❑	3 ❑	4 ❑	4	How often do you have a choice between healthcare centers?	1 ❑	2 ❑	3 ❑	4 ❑
1 ❑	2 ❑	3 ❑	4 ❑	5	How often do you have a choice between health care providers in that healthcare center?	1 ❑	2 ❑	3 ❑	4 ❑
1 ❑	2 ❑	3 ❑	4 ❑	6	How often do you have the opportunity to see a specialist, if you wish to?	1 ❑	2 ❑	3 ❑	4 ❑
**Poor**	**Fair**	**Good**	**Excellent**	**B: Communication**<br>**In relation to your usual medical providers:**		**Never**	**Sometimes**	**Usually**	**Always**
1 ❑	2 ❑	3 ❑	4 ❑	7	How often do you have the **opportunity to ask questions** about your conditions during the consultation?	1 ❑	2 ❑	3 ❑	4 ❑
1 ❑	2 ❑	3 ❑	4 ❑	8	How often do **care providers listen carefully** to your concerns and answer your questions?	1 ❑	2 ❑	3 ❑	4 ❑
**Poor**	**Fair**	**Good**	**Excellent**			**Poor**	**Fair**	**Good**	**Excellent**
1 ❑	2 ❑	3 ❑	4 ❑	9	How would you rate the **amount of information** you have received from the care providers?	1 ❑	2 ❑	3 ❑	4 ❑
1 ❑	2 ❑	3 ❑	4 ❑	10	How would you rate the amount of information you have received from the care providers about health system facilities?	1 ❑	2 ❑	3 ❑	4 ❑
1 ❑	2 ❑	3 ❑	4 ❑	11	How would you rate the clarity of information you have received from the care providers?	1 ❑	2 ❑	3 ❑	4 ❑
**Poor**	**Fair**	**Good**	**Excellent**		**C: Autonomy**	**Poor**	**Fair**	**Good**	**Excellent**
1 ❑	2 ❑	3 ❑	4 ❑	12	How would you rate the support from your care	1 ❑	2 ❑	3 ❑	4 ❑

					providers in making choices?				
1 ❑	2 ❑	3 ❑	4 ❑	13	How would you rate the usefulness of information you have received from the care providers to make a choice?	1 ❑	2 ❑	3 ❑	4 ❑
1 ❑	2 ❑	3 ❑	4 ❑	14	How would you rate the opportunity to select or refuse treatment without pressure from care providers?	1 ❑	2 ❑	3 ❑	4 ❑
1 ❑	2 ❑	3 ❑	4 ❑	15	How would you rate the extent to which your care providers sought your consent before testing or starting treatment'	1 ❑	2 ❑	3 ❑	4 ❑
**Poor**	**Fair**	**Good**	**Excellent**		**D: Support group**	**Poor**	**Fair**	**Good**	**Excellent**
1 ❑	2 ❑	3 ❑	4 ❑	16	How would you rate the opportunity' to be supported by a group of pregnant women?	1 ❑	2 ❑	3 ❑	4 ❑
1 ❑	2 ❑	3 ❑	4 ❑	17	How would you rate the opportunity to share your information and experiences with other pregnant women?	1 ❑	2 ❑	3 ❑	4 ❑
1 ❑	2 ❑	3 ❑	4 ❑	18	How would you rate the opportunity to receive useful information from other pregnant women who have knowledge and skill about different aspects of their condition?	1 ❑	2 ❑	3 ❑	4 ❑
1 ❑	2 ❑	3 ❑	4 ❑	19	How would you rate the opportunity to be educated by experts (e.g. gynecologist, midwife, ...) in your group with other pregnant women?	1 ❑	2 ❑	3 ❑	4 ❑
**Poor**	**Fair**	**Good**	**Excellent**		**E: Continuity**	**Poor**	**Fair**	**Good**	**Excellent**
1 ❑	2 ❑	3 ❑	4 ❑	20	How would you rate the opportunity to see your regular **healthcare provider** in your routine visits?	1 ❑	2 ❑	3 ❑	4 ❑
1 ❑	2 ❑	3 ❑	4 ❑	21	How would you rate the opportunity to see the same **SPECIALIST** or **GP** that you have seen before?	1 ❑	2 ❑	3 ❑	4 ❑
1 ❑	2 ❑	3 ❑	4 ❑	22	how would you rate the **continuity and consistency of information** about diabetes (e.g. diet, prevention and treatment) that you received from different care providers?	1 ❑	2 ❑	3 ❑	4 ❑
**Poor**	**Fair**	**Good**	**Excellent**		**F: Quality of basic amenities**	**Poor**	**Fair**	**Good**	**Excellent**
1 ❑	2 ❑	3 ❑	4 ❑	23	How would you rate the cleanliness of health care providers?	1 ❑	2 ❑	3 ❑	4 ❑
1 ❑	2 ❑	3 ❑	4 ❑	24	How would you rate the cleanliness of health care facilities you usually attend?	1 ❑	2 ❑	3 ❑	4 ❑
1 ❑	2 ❑	3 ❑	4 ❑	25	How would you rate the adequacy of furniture in health care facilities you usually attend?	1 ❑	2 ❑	3 ❑	4 ❑
1 ❑	2 ❑	3 ❑	4 ❑	26	How would you rate the cleanliness of toilets in health care facilities you usually attend?	1 ❑	2 ❑	3 ❑	4 ❑
**Poor**	**Fair**	**Good**	**Excellent**		**G: Dignity**	**Poor**	**Fair**	**Good**	**Excellent**
1 ❑	2 ❑	3 ❑	4 ❑	27	How would you rate the emotional support given by your usual care providers'?	1 ❑	2 ❑	3 ❑	4 ❑
1 ❑	2 ❑	3 ❑	4 ❑	28	Your beliefs and culture were respected by care providers:	1 ❑	2 ❑	3 ❑	4 ❑
1 ❑	2 ❑	3 ❑	4 ❑	29	How would you rate the amount of information given your family or someone close to you?	1 ❑	2 ❑	3 ❑	4 ❑
**Never**	**Sometimes**	**Usually**	**Always**			**Never**	**Sometimes**	**Usually**	**Always**
1 ❑	2 ❑	3 ❑	4 ❑	30	Service providers encourage to express your feelings and concerns about your pregnancy:	1 ❑	2 ❑	3 ❑	4 ❑
1 ❑	2 ❑	3 ❑	4 ❑	31	Respect was shown for your privacy during treatment and examinations:	1 ❑	2 ❑	3 ❑	4 ❑
**Never**	**Sometimes**	**Usually**	**Always**		**H: Timeliness and prompt attention**	**Never**	**Sometimes**	**Usually**	**Always**
1 ❑	2 ❑	3 ❑	4 ❑	32	Does your healthcare provider keep you in the waiting room for more than 15 minutes?	1 ❑	2 ❑	3 ❑	4 ❑
1 ❑	2 ❑	3 ❑	4 ❑	33	Does your GP keep you in the waiting room for more	1 ❑	2 ❑	3 ❑	4 ❑

					than IS minutes'?				
						**Poor**	**Fair**	**Good**	**Excellent**
1 ❑	2 ❑	3 ❑	4 ❑	34	How would you rate the time you have to wait until your next appointment with your healthcare provider?	1 ❑	2 ❑	3 ❑	4 ❑
1 ❑	2 ❑	3 ❑	4 ❑	35	How would you rate the time you have to wait until your next appointment with gynecologist?	1 ❑	2 ❑	3 ❑	4 ❑
1 ❑	2 ❑	3 ❑	4 ❑	36	How would you rate the case of making a new appointment with gynecologist (in general)?	1 ❑	2 ❑	3 ❑	4 ❑
**Poor**	**Fair**	**Good**	**Excellent**		**I: Safety**	**Poor**	**Fair**	**Good**	**Excellent**
1 ❑	2 ❑	3 ❑	4 ❑	37	How would you rate the explanation of the purpose of the medication, tests, or treatments in a way you could understand?	1 ❑	2 ❑	3 ❑	4 ❑
1 ❑	2 ❑	3 ❑	4 ❑	38	How would you rate the side effects of medications in a way you could understand?	1 ❑	2 ❑	3 ❑	4 ❑
1 ❑	2 ❑	3 ❑	4 ❑	39	How would you rate the avoid any (physical-mental) harm to pregnant mothers while receiving healthcare?	1 ❑	2 ❑	3 ❑	4 ❑
**Poor**	**Fair**	**Good**	**Excellent**		**J: prevention**	**Poor**	**Fair**	**Good**	**Excellent**
1 ❑	2 ❑	3 ❑	4 ❑	40	How would you rate the explanation of warning signs recording complications during pregnancy and high-risk delivery symptoms in a way you could understand?	1 ❑	2 ❑	3 ❑	4 ❑
1 ❑	2 ❑	3 ❑	4 ❑	41	How would you rate the explanation about the medication side effects by your GP/healthcare provider?	1 ❑	2 ❑	3 ❑	4 ❑
1 ❑	2 ❑	3 ❑	4 ❑	42	How would you rate the explanation and training about self-care educations during pregnancy?	1 ❑	2 ❑	3 ❑	4 ❑
**Poor**	**Fair**	**Good**	**Excellent**		**K: Availability**	**Poor**	**Fair**	**Good**	**Excellent**
1 ❑	2 ❑	3 ❑	4 ❑	43	How would you rate the geographical accessibility of health care facilities for pregnant women?	1 ❑	2 ❑	3 ❑	4 ❑
1 ❑	2 ❑	3 ❑	4 ❑	44	How would you rate the financial accessibility of healthcare for pregnant women (affordability)?	1 ❑	2 ❑	3 ❑	4 ❑
1 ❑	2 ❑	3 ❑	4 ❑	45	How would you rate the relevance of services with beliefs and faith of pregnant women?	1 ❑	2 ❑	3 ❑	4 ❑
**Poor**	**Fair**	**Good**	**Excellent**		**L: Confidentiality**	**Poor**	**Fair**	**Good**	**Excellent**
1 ❑	2 ❑	3 ❑	4 ❑	46	How would you rate the confidentiality of information provided to service providers?	1 ❑	2 ❑	3 ❑	4 ❑
1 ❑	2 ❑	3 ❑	4 ❑	47	How would you rate the confidentiality of information registered in medical record (laboratory tests, …) ?	1 ❑	2 ❑	3 ❑	4 ❑

Customer quality measure

Below are 19 statements that people sometimes make when they talk about their health. Please indicate how much you agree or disagree with each statement as it applies to you personally by marking your answer. Your answer should be what is true for you and not just what you think the doctor wants you to say. If the statement does not apply to you. Please mark "Not applicable".

	performance				
	**Disagreed strongly**	**Disagree**	**Not applicable**	**Agree**	**Agree strongly**
1- When all is said and done, I am the person who is responsible for managing my health.	1 ❑	2 ❑	3 ❑	4 ❑	5 ❑
2- Taking an active role in my own health care is the most important factor in determining my health and ability to function	1 ❑	2 ❑	3 ❑	4 ❑	5 ❑
3- I am confident that I can take actions that will help prevent or minimize some symptoms or problems associated with my health.	1 ❑	2 ❑	3 ❑	4 ❑	5 ❑
4- I know what each of my prescribed medications does.	1 ❑	2 ❑	3 ❑	4 ❑	5 ❑
5- I am confident that I can tell when I need to get medical care and when I can handle a health problem myself.	1 ❑	2 ❑	3 ❑	4 ❑	5 ❑
6- I am confident that I can tell my doctor concerns I have even when he or she does not ask.	1 ❑	2 ❑	3 ❑	4 ❑	5 ❑
7- I am confident that I can follow through on medical treatments I may need to do at home	1 ❑	2 ❑	3 ❑	4 ❑	5 ❑
8- I understand the nature and causes of my health problems.	1 ❑	2 ❑	3 ❑	4 ❑	5 ❑
9- I know the different medical treatment options available for my health conditions.	1 ❑	2 ❑	3 ❑	4 ❑	5 ❑
10- I have been able to maintain the lifestyle changes that I have made for my health.	1 ❑	2 ❑	3 ❑	4 ❑	5 ❑
11- I know how to prevent problems with my health.	1 ❑	2 ❑	3 ❑	4 ❑	5 ❑
12- 1 am confident I can figure out solutions when new situations or problems arise with my health.	1 ❑	2 ❑	3 ❑	4 ❑	5 ❑
13- 1 am confident that I can maintain lifestyle changes, like diet and exercise.	1 ❑	2 ❑	3 ❑	4 ❑	5 ❑
14- I know how to prevent the problems threat my health.	1 ❑	2 ❑	3 ❑	4 ❑	5 ❑
15- I am confident that I'm able to find a solution to my new health problems	1 ❑	2 ❑	3 ❑	4 ❑	5 ❑
16- I have the opportunity to actively participate in self-care or cooperation with the health team.	1 ❑	2 ❑	3 ❑	4 ❑	5 ❑
17- I am able to work with the members of the health team (health care staff) to improve my health and improve the quality of my service.	1 ❑	2 ❑	3 ❑	4 ❑	5 ❑
18- I am confident that I can make changes to my way of life (such as diet and exercise), even in times of stress, anxiety and difficult conditions.	1 ❑	2 ❑	3 ❑	4 ❑	5 ❑
19- I am confident that I can keep my lifestyle changes (diet, exercise, etc.) even in case of financial constraints.	1 ❑	2 ❑	3 ❑	4 ❑	5 ❑

Part III: demographics

A: Individual profile

1. Birthday of the year ...... 2. Place of Birth ...... 3. Current Accommodation........ 4. Language ......

2. Specify the type of job:

Self-employed ☐ employee ☐ hired ☐ temporary worker ☐
Student ☐ retirement ☐ other (with name)..............

3. Are you under certain health insurance coverage? Yes ☐ No ☐

If the answer is yes, specify the type of insurance:

Health Insurance ☐ Supplementary Insurance ☐ Social Security Insurance ☐

Armed Forces Insurance Insurance ☐ Committee Self-Employed Insurance ☐

Rural Insurance ☐ Others (with name).......................

4. Education level:

Elementary ☐ Illiterate ☐ High School ☐ Diploma ☐
Bachelor's Degree ☐ Master's degree ☐ Ph.D ☐

(B) The status of pregnancy

1. What is the current pregnancy of several pregnancies? ....

2. How many kids do you have now? ....

3. How many times have you given birth? ..... Normal Delivery ☐ Caesarean section ☐

4. Have you had a history of abortion? ...... Several times? ......

5. Have you ever been dead? .... Several times? ......

6. Was the current pregnancy planned? Yes ☐ No ☐

7. Where did you go for prenatal care?

Health House ☐ Health Center ☐ Obstetrician ☐ Other gynecologist ☐
(please note) ....

8. Are you regularly taken care of? Yes ☐ No ☐

(C) Smoking Status

1. Have you ever smoked? Yes ☐ No ☐ if yes answer:

2. At what age did you have the first cigar? ....

3. Do you smoke now? Yes no if yes answer:

4. How many threads do you smoke a day? ....

5. If you smoke but did not take it now, at what age did you quit smoking? .....

Thank you for your cooperation and contribution to the above research project

Please, "If you have not heard anything, or if you would like to make a new massage about your pregnancy experience, note here:

....
....
....
....
....
....
....
....
....
....
....
....
....
....

Please, "If you have a comment on the questionnaire and its questions, please note here:

....
....
....
....
....
....
....
....
....
....
....
....
....
....
