# Supplementary material for: Effects of customer self-audit on the quality of maternity care in Tabriz: A cluster-randomized controlled trial
Source: PLoS One. 2018 Oct 11;13(10):e0203255. doi: 10.1371/journal.pone.0203255 (PMC6181295; doi:10.1371/journal.pone.0203255)
Supplement: S8 File — (PDF) [file pone.0203255.s008.pdf]

پرسشنامه بررسی کیفیت مراقبت های دوران بارداری

راهنمای پرسشنامه

قسمت اول: کیفیت خدمت (Service Quality)

- برای پاسخ به سوالات از یک خودکار یا مداد استفاده کنید. میتوانید اشتباهاتتان را پاک کرده و یا تصحیح کنید.
- اگر مطمئن نیستید کدام گزینه را انتخاب کنید، نزدیکترین جوابی که فکر می کنید صحیح است را علامت بزنید.
- لطفا نظرات خود و یا اطلاعات دیگری را که می خواهید اضافه کنید، در صفحه آخر بنویسید.

اهمیت:

در زیر لیستی از موارد مهم و کم اهمیت در مورد خدمتی که به شما ارائه می شود وجود دارد، لطفا در باره هر مورد مشخص کنید چقدر برایتان مهم است. در بین گزینه ها جواب غلط و یا درست وجود ندارد. قبل از پاسخ به سوالات لطفا" مثال زیر را به دقت مطالعه کنید.

مثال: داشتن حق انتخاب بین مراکز ارائه کننده خدمات بهداشتی درمانی (خدمات درمانی)

۱- مهم نیست ۲- نسبتا مهم است ۳- مهم است ۴- خیلی مهم است

اگر شما در مثال مورد اول را علامت بزنید معنی آن، این است که شما فکر می کنید بین واحدهای مختلف ارائه مراقبت تفاوت وجود ندارد، بنابراین شما «مهم نیست» را علامت زده اید.

عملکرد:

در این زمینه ما می خواهیم تجربیات شما را از کیفیت خدمت (جنبه غیر پزشکی مراقبت ها) بدانیم. لطفا با دقت مثال را بخوانید و به همه سوالات پاسخ دهید.

مثال: آیا در صورت نیاز می توانید به متخصص مراجعه کنید (امکان گرفتن نوبت را دارید)؟

۱- هیچ وقت ۲- گاهی اوقات ۳- اغلب ۴- همیشه

اگر شما در مثال بالا مورد دوم را علامت بزنید ، به این معنی است که شما به این فکر کرده اید که شانس کمی برای مراجعه به متخصصی را داشتید که حتما می خواستید به او مراجعه کنید، بنابرین شما «گاهی اوقات» را علامت زده اید.

توجه:

لازم به ذکر است که شرکت در تحقیق فوق و تکمیل پرسشنامه کاملا" اختیاری بوده و شما مختارید در هر زمان که اراده کنید از مشارکت در تحقیق انصراف بدهید. اطلاعات ارائه شده از جانب شما کاملا" محرمانه بوده و هیچ فرد دیگری غیر از اعضای تیم تحقیق به آن دسترسی نخواهد داشت. ضمنا" همکاری شما در این امر به معنی رضایت آگاهانه شما برای مشارکت در تحقیق تلقی خواهد شد.

سئوالات و گویه های ابعاد کیفیت خدمت

الف: انتخاب ارائه کننده

۱ : برای نیاز های مراقبتی معمول دوران بارداری شما به کدام ارائه کننده خدمات بهداشتی و درمانی مراجعه می کنید؟(شما می توانید بیش از یک مورد را انتخاب کنید)

پزشک عمومی یا خانواده	☐ ۱	ماما یا کادر بهداشت خانواده	☐ ٤
متخصص زنان	☐ ۲	پرستار	☐ ٥
سایر متخصصین	☐ ۳	ارائه کنندگان نامشخص	☐ ٦

سایر(لطفا مشخص کنید.................................) ☐ ۷

۲- آیا شما در طول بارداری فعلی فقط به یک ارائه کننده خدمت خاص (پزشک ،کادر بهداشت خانواده و....) مراجعه کرده اید ؟

بلی ☐ خیر ☐

۳- آیا شما در طول بارداری فعلی فقط به یک متخصص زنان مشخص مراجعه کرده اید ؟ بلی ☐ خیر ☐

عملکرد				گویه	اهمیت			
هیچ وقت	گاهی	اغلب	همیشه		مهم نیست	نسبتا" مهم	مهم	خیلی مهم
				**الف- انتخاب ارائه کننده خدمت**				
				۴- داشتن حق انتخاب بین واحدهای ارائه کننده خدمات بهداشتی درمانی				
				۵- داشتن حق انتخاب بین ارائه کنندگان مراقبت در یک واحد ارائه کننده خدمات بهداشتی درمانی				
				۶- داشتن حق انتخاب برای ویزیت توسط متخصصی (موارد ارجاع) که شما می خواهید				
				**ب- ارتباط و تعامل**				
				۷- امکان پرسیدن سوالات در مورد وضعیت خودتان (از پزشک) در طی ویزیت				
				۸- با دقت گوش کردن ارائه دهندگان خدمات به نگرانی شما و پاسخ به سوالات شما در یک حالت دوستانه				
ضعیف	متوسط	خوب	عالی	۹- میزان اطلاعاتی که از ارائه کنندگان خدمت در مورد مراقبت های دوران بارداری دریافت می کنید				
				۱۰- میزان اطلاعاتی که از ارائه کنندگان خدمت در مورد محل های ارائه خدمات دریافت می کنید				
				۱۱ - کیفیت (شفاف بودن) اطلاعاتی که از ارائه کنندگان خدمت در مورد دوران بارداری دریافت می کنید.				
				**ج- داشتن اختیار**				
				۱۲- دریافت اطلاعات کافی از ارائه کنندگان خدمت برای تصمیم گیری در باره بارداری				
				۱۳- مشارکت با ارائه کنندگان خدمت در تصمیم گیری				
				۱۴- قدرت انتخاب یا رد کردن درمان یا مراقبت (بدون فشار و اجبار از سوی ارائه کننده گان خدمت)				
				۱۵- اخذ رضایت از شما (توسط ارائه کنندگان خدمات) قبل از انجام آزمایشات و یا شروع درمان				
				**د- گروههای حمایتی**				
				۱۶- امکان حمایت شدن توسط سایر زنان باردار (افرادی با شرایط مشابه شما)				
				۱۷- امکان در میان گذاشتن نظرات، اطلاعات و تجربیات شما با سایر زنان باردار				
				۱۸- امکان دریافت تجربیات و اطلاعات مفید از دیگر مادران باردار که دارای دانش و مهارتهایی در مورد جنبه های مختلف دوران بارداری هستند				
				۱۹- امکان استفاده از آموزشهای متخصصین (به عنوان مثال:متخصص زنان ، متخصص تغذیه ،...) در گروهی (جلسات آموزشی) با حضور سایر زنان باردار				
				**ه- استمرار خدمات**				
				۲۰- امکان دیدن مسئول دائمی مراقبت هایتان در ویزیت های معمول شما				
				۲۱- امکان ویزیت همان پزشک عمومی یا متخصص که شما قبلا" به او مراجعه کرده اید				
				۲۲- استمرار و پیوستگی و ارتباط اطلاعات دریافتی شما از ارائه کننده گان متفاوت خدمات (کادر بهداشت خانواده،ماما،پزشک و...)				

عملکرد				گویه	اهمیت			
ضعیف	متوسط	خوب	عالی		مهم نیست	نسبتا" مهم	مهم	خیلی مهم
				**و- کیفیت و تسهیلات و امکانات اولیه**				
				۲۳- تمیزی و رعایت بهداشت فردی پرسنلی که شما به آنها مراجعه می کنید				
				۲۴- تمیزی و پاکیزگی درمانگاه (مرکز ارائه خدمات بهداشتی درمانی) که شما معمولا به آنجا مراجعه می کنید				
				۲۵- مناسب بودن و کافی بودن وسایل، میز و صندلی در محل درمانگاه (مرکز ارائه خدمات بهداشتی درمانی) که شما معمولا به آنجا مراجعه می کنید				
				۲۶- تمیزی دستشوئی ها و توالت های مراکزی که معمولا به آنجا مراجعه می کنید				
				**ز- احترام**				
				۲۷- حمایت عاطفی ارائه کنندگان خدمت نسبت به شما				
				۲۸- احترام به باورها و فرهنگ شما به وسیله ارائه کنندگان خدمات				
هیچ وقت	گاهی	اغلب	همیشه	۲۹- تشویق ارائه کنندگان خدمات به بیان احساس و نگرانی های شما درباره بارداریتان				
				۳۰- احترام به حریم خصوصی شما در طی مراقبت، درمان و انجام آزمایشات				
				۳۱- میزان اطلاعات داده شده به خانواده یا نزدیکان شما، برای کمک به مراقبت شما				
				**ح- به مو قع بودن و توجه فوری**				
				۳۲- انتظار بیش از ۱۵ دقیقه در اتاق انتظار ارائه کننده مراقبت های شما (قبل از مراقبت)				
				۳۳- انتظار بیش از ۱۵ دقیقه در اتاق انتظار پزشک عمومی یا پزشک متخصص				
ضعیف	متوسط	خوب	عالی	۳۴- انتظار تا نوبت ملاقات بعدی با ارائه کننده مراقبت ها				
				۳۵- انتظار تا نوبت ملاقات بعدی با پزشک متخصص زنان خودتان				
				۳۶- سهولت نوبت گرفتن از متخصص زنان				
				**ط - ایمنی**				
				۳۷- توضیح درباره هدف ازتجویز داروها، انجام آزمایشات یا درمان ها (قابل فهم برای شما)				
				۳۸- توضیح درباره عوارض جانبی داروها به وسیله پزشک شما یا دیگران				
				۳۹- اجتناب از هر گونه آسیب (جسمی- روحی) به مادران باردار در حین ارائه خدمت				
				**ی- پیشگیری**				
				۴۰- توضیح ارائه شده توسط ارائه دهندگان خدمات در مورد علائم هشدار مرتبط با عوارض دوران بارداری و نشانه های زایمان پر خطر				
				۴۱- توضیح درباره اقداماتی که از بروز عوارض جلوگیری می کند				
				۴۲- توضیح و آموزش در مورد خود مراقبتی (self care) طی دوران بارداری				

**ک- دسترسی**								
				۴۳- دسترسی جغرافیایی به درمانگاه، بیمارستان و یا محل های ارائه مراقبتهای بهداشتی درمانی				
				۴۴- دسترسی اقتصادی و مالی (توان مالی برای پرداخت هزینه های مراقبت های دوران بادرای)				
				۴۵- تطابق خدمات با باورها و عقاید و اداب و رسوم				
**ل- اعتماد**								
				۴۶- رازداری و اطمینان از محرمانه بودن اطلاعاتی که به ارائه کنندگان خدمات می دهید				
				۴۷- اطمینان از محرمانه بودن خدمات موجود در پرونده پزشکی(نتایج آزمایشات و ...)				

قسمت سوم : کیفیت مشتری (Customer Quality)

در این قسمت ۱۹ موضوعی را که ممکن است در امر سلامتی مردم نقش داشته باشد ذکر کرده ایم. در صورتیکه هر یک از موضوعات به شخص شما مربوط می شود، با انتخاب پاسخ مناسب، مشخص کنید تاچه اندازه با موضوع مورد نظر موافق یا مخالف هستید. پاسخ شما باید در بر گیرنده آن چیزی باشد که در مورد شما صدق می کند

ردیف	گویه های کیفیت مشتری	کاملاً موافقم	موافقم	موردی نداشته ام	مخالفم	کاملاً مخالفم
۱.	**بعد از انجام همه اقدامات بهداشتی درمانی از سوی کادر پزشکی، نهایتاً من مسئول حفظ سلامتی خود هستم.**	☐	☐	☐	☐	☐
۲.	**داشتن نقش فعال در مراقبت های سلامتي خودم عامل بسیار مهمی در تعیین سلامتی و توان عملکردی ام می باشد.**	☐	☐	☐	☐	☐
۳.	**من مطمئن هستم می توانم اقداماتی را انجام دهم که منجر به پیشگیری یا کاهش خطرات مرتبط با سلامتی ام گردد.**	☐	☐	☐	☐	☐
٤.	**من می دانم هر کدام از داروهای تجویز شده برایم به خاطر چیست .**	☐	☐	☐	☐	☐
٥.	**من می دانم مراقبت ها و اقدامات پزشکی ارائه شده برایم به خاطر چیست.**	☐	☐	☐	☐	☐
٦.	**من اطمینانَ دارم می توانم تشخیص بدهم که چه موقع به خدمات پزشکی (مراقبت های بهداشتی و درمانی) نیاز دارم و چه موقع میتوانم مشکلاتم را خودم (بدون مراجعه به پزشک) حل کنم.**	☐	☐	☐	☐	☐
۷.	**من اطمینان دارم می توانم نگرانی های مرتبط با سلامتیم را به پزشک معالجم اعلام کنم، حتی زمانی که او از من نپرسد .**	☐	☐	☐	☐	☐
۸.	**من اطمینان دارم در صورت نیاز به مراقبتهای بهداشتی در منزل، خود میتوانم آن را مطابق دستور پزشک یا کارکنان بهداشتی انجام دهم.**	☐	☐	☐	☐	☐
۹.	**من منشاء و دلایل مشکلات مرتبط با سلامتیم را می دانم.**	☐	☐	☐	☐	☐
۱۰.	**من از سیر مشکلاتم یا پیش آگهی آنها به طور کامل اطلاع دارم.**	☐	☐	☐	☐	☐
۱۱.	**من از انواع روش های مراقبتي و درمانی قابل انجام در مورد دوران بارداری مطلع هستم.**	☐	☐	☐	☐	☐
۱۲.	**من خود را عضوی از تیم سلامت می دانم و باور دارم که مشارکت و همکاری من در فرایند مراقبت و درمان باعث بهبود کیفیت خدمات و ارتقای سلامتی خودم خواهد شد.**	☐	☐	☐	☐	☐
۱۳.	**من می توانم تغییراتی را که برای تامین سلامتی، در روش زندگی خود انجام داده ام، حفظ کنم .**	☐	☐	☐	☐	☐
۱٤.	**من میدانم چگونه ازمسائل و مشکلات مرتبط با سلامت خود پیشگیری کنم.**	☐	☐	☐	☐	☐
۱٥.	**من اطمینان دارم قادر به پیدا کردن راه حل جهت رفع مشکلات جدید سلامتی ام هستم.**	☐	☐	☐	☐	☐
۱٦.	**من عملاً امکان مشارکت فعال در مراقبت از خود و یا همکاری با تیم سلامت را دارم.**	☐	☐	☐	☐	☐
۱۷.	**من قادر هستم با اعضای تیم سلامت (کارکنان بهداشتی و درمانی) در ارائه خدمات همکاری کرده و با بهبود کیفیت خدمت ارائه شده سلامت خود را ارتقا دهم.**	☐	☐	☐	☐	☐
۱۸.	**من اطمینان دارم که می توانم تغییراتی را که در روش زندگی ام داده ام (مانند رژیم غذایی و ورزش) حتی در مواقع استرس، اضطراب و شرایط سخت نیز حفظ کنم.**	☐	☐	☐	☐	☐
۱۹.	**من اطمینان دارم می توانم حتی در صورت محدودیت های مالی نیز تغییرات بعمل آورده در شیوه زندگی ام (رژیم غذایی، ورزش و...) را حفظ کنم.**	☐	☐	☐	☐	☐

قسمت چهارم: اطلاعات فردی

الف: مشخصات فردی

۱.تاریخ تولد سال...... ۲ . محل تولد...... ۳. محل اقامت فعلی........ ۴. زبان......

۷. نوع شغل را مشخص کنید:
☐ کارمند ☐ شغل آزاد ☐ استخدام موقتی ☐ خانه دار
☐ بازنشسته ☐ دانشجو سایر موارد.(با ذکر نام).............

۸. آیا تحت پوشش بیمه درمانی خاصی هستید: بلی ☐ خیر ☐

در صورتی که جواب بلی است نوع بیمه را مشخص کنید:
☐ بیمه خدمات درمانی ☐ بیمه های مکمل ☐ بیمه تامین اجتماعی ☐ بیمه نیروی مسلح
☐ بیمه کمیته امداد ☐ بیمه خویش فرما ☐ بیمه روستایی
سایر(با ذکر نام)......................

۹. سطح تحصیلات:
☐ بیسواد ☐ ابتدایی ☐ راهنمایی ☐ دبیرستان ☐ دیپلم
☐ فوق دیپلم ☐ لیسانس ☐ فوق لیسانس ☐ دکتری ☐ فوق دکتري

ب: وضعیت بارداری

۱. حاملگی فعلی چندمین حاملگی است؟ ....
۲. اکنون چند بچه دارید؟ ....
۳. چند بار زایمان کرده اید؟..... طبیعی ☐ سزارین ☐
۴. آیا سابقه سقط[۱] داشته اید ؟ ...... چند بار؟ ......
۵. آیا مرده زایی[۲] داشته اید؟ .... چند بار؟ ......
۶. آیا حاملگی فعلی برنامه ریزی شده بود؟ بلی ☐ خیر ☐
۷. برای مراقبت دوران بارداری به کجا مراجعه کرده اید؟
☐ خانه بهداشت ☐ مرکز بهداشتی و درمانی ☐ مطب مامایی ☐ متخصص زنان
سایر(باذکرنام)................................
۸. آیا به طور مرتب مراقبت شده اید؟ بلی ☐ خیر ☐

ج: بررسی استعمال سیگار

۱. آیا تاکنون سیگار کشیده اید؟ بلی ☐ خیر ☐ در صورت بلی بودن جواب:
۲. اولین سیگار را در چه سنی کشیده اید؟ ....
۳. آیا الآن سیگار می کشید؟ بلی ☐ خیر ☐ در صورت بلی بودن جواب:
۴. چند نخ در روز سیگار می کشید؟ ....
۵. اگر سیگارمی کشیدید ولی الان نمی کشید،در چه سنی سیگار را ترک کرده اید؟.....

[۱] حاملگی ناموفق قبل از ۲۰ هفتگی
[۲] حاملگی نا موفق بعد از ۲۰ هفتگی

با تشکرصمیمانه از همکاری و مشارکت شما در انجام پروژه تحقیقاتی فوق

لطفا"؛ در صورتیکه مطلب ناگفته ای مانده است یا تمایل دارید مطلب جدیدی در بار ه تجربه خود از دوران بارداری را مطرح کنید، در این قسمت یادداشت فرمائید:

....
....
....
....
....
....
....
....
....
....
....
....
....
....
....

لطفا" در صورتیکه نظری در باره پرسشنامه و سئوالات آن دارید در این قسمت یادداشت فرمائید:

....
....
....
....
....
....
....
....
....
....
....
....
....
....
....
